# Supplementary material for: Prediction of cellular morphology changes under perturbations with a transcriptome-guided diffusion model
Source: Nat Commun. 2025 Sep 2;16:8210. doi: 10.1038/s41467-025-63478-z (PMC12405515; doi:10.1038/s41467-025-63478-z)
Supplement: Supplementary file 1 — supplementary_information [file 41467_2025_63478_MOESM1_ESM.pdf]

Supplementary Information for: Prediction of cellular morphology changes under perturbations with a transcriptome-guided diffusion model

Xuesong Wang†[1,2], Yimin Fan†[1,2], Yucheng Guo†[1], Chenghao Fu[2], Kinhei Lee[2], Khachatur Dallakyan[2], Yaxuan Li[2], Qijin Yin[1], Yu Li‡[2,3], and Le Song‡[1,4]

[1]BioMap Research, California, USA
[2]Department of Computer Science and Engineering, The Chinese University of Hong Kong, Hong Kong SAR, China
[3]The Chinese University of Hong Kong Shenzhen Research Institute, Shenzhen, China
[4]Mohamed bin Zayed University of Artificial Intelligence, Abu Dhabi, UAE
†Equal contribution
‡Corresponding authors. Email: yuli@cuhk.edu.hk and le.song@mbzuai.ac.ae

Contents

A Supplementary Notes **3**
A.1 Baseline Methods 3
A.2 Criteria for choosing the images for visualization 4
A.3 Analysis of the impact of the quality of the control morphology images on I2I mode performance as well as practical tips 4
A.4 Analyzing the applicability of MorphDiff on OOD MOAs and targets 4

B Supplementary Tables **6**

C Supplementary Figures **13**

List of Tables

1 The CellProfiler channel name refers to the name assigned by the CellProfiler to each channel . . 6
2 Comparison of MorphDiff with other related tools 6
3 Detailed results of the Maximum Mean Discrepancy (MMD) test on the CDRP dataset (Repeat 10 times). It is a two-sided test and adjustments for multiple comparisons are not applicable. 7
4 Detailed results of the Energy Distance test on the CDRP dataset (Repeat 10 times). It is a two-sided test and adjustments for multiple comparisons are not applicable. 7
5 Detailed results of the Maximum Mean Discrepancy (MMD) test on the JUMP dataset (Repeat 10 times). It is a two-sided test and adjustments for multiple comparisons are not applicable. 8
6 Detailed results of the Energy Distance test on the JUMP dataset (Repeat 10 times). It is a two-sided test and adjustments for multiple comparisons are not applicable. 8
7 Detailed results of the Maximum Mean Discrepancy (MMD) on the LINCS Target leave-one-out set (Repeat 10 times). It is a two-sided test and adjustments for multiple comparisons are not applicable. 9
8 Detailed results of the Energy Distance Test on the LINCS Target leave-one-out set (Repeat 10 times). It is a two-sided test and adjustments for multiple comparisons are not applicable. 10
9 Detailed results of the Maximum Mean Discrepancy on the LINCS MOA leave-one-out set (Repeat 10 times). It is a two-sided test and adjustments for multiple comparisons are not applicable. 11
10 Detailed results of the Energy Distance Test on the LINCS MOA leave-one-out set (Repeat 10 times). It is a two-sided test and adjustments for multiple comparisons are not applicable. 12

List of Figures

1 Visualization of the generated images from each baseline method as well as the ground-truth from the JUMP dataset (*CARD11*, *CDKN11* and *DVL1*) 13
2 Visualization of the generated images from each baseline method as well as the ground-truth from the JUMP dataset (*MAP3K2*, *NOTCH2* and *PIK3C*.) 14
3 Visualization of the generated images from each baseline method as well as the ground-truth from the JUMP dataset (*PRKCZ*, *PTEN* and *SMAD5*.) 15
4 UMAP visualization of the generated cell morphology CellProfiler feature on 18 genetic perturbations for IMPA and MorphDiff(G2I). 16
5 UMAP visualization of the generated cell morphology CellProfiler feature on 18 genetic perturbations for IMPA and MorphDiff(I2I). 17
6 Distribution of CellProfiler features between ground-truth, IMPA-generated, and MorphDiff-generated morphology on *JUN*, *CDK2* and *STAT3* genetic perturbation. 18
7 Distribution of CellProfiler features between ground-truth, IMPA-generated, MorphDiff(G2I)-generated and MorphDiff(I2I)-generated morphology on *RAC1*, *MCL1* and *ACVR1B* genetic perturbation. 19
8 Additional evaluation using various tests for Figure 2e in the manuscript. 20
9 Correlation heatmap between the gene expression and the CellProfiler Features from the ground-truth cell morphology. 21
10 Correlation heatmap between the gene expression and the CellProfiler Features from the predicted cell morphology of MorphDiff. 22
11 Correlation heatmap between the gene expression and the CellProfiler Features from the predicted cell morphology of IMPA. 23
12 The heatmap of the correlation between the CellProfiler features of ground-truth morphology, MorphDiff(I2I)-generated morphology, and IMPA-generated morphology with L1000 gene expression. 24
13 Leave-one-out validation performance on 10 MOAs and 10 targets on the A549 cell line. 24
14 Additional evaluation on the R^2 score between the ground-truth and generated CellProfiler features on the CDRP dataset (For Figure 3 in the manuscript) 25
15 Additional evaluation on the R^2 score between the ground-truth and generated CellProfiler features on the CDRP dataset (For Figure 3 in the manuscript) 26
16 Additional evaluation analysis on the LINCS dataset (For Figure 3 in the manuscript) 27
17 Drug structures involved in the analysis of Figure 3 in the manuscript 28
18 MorphDiff predicts morphological changes on the target level. 29
19 MorphDiff predicts morphological changes on the target level. 30
20 Ablation study on the number of k in the top k MOA retrieval 31
21 Performance of MOA matching for the methods according to two metrics 32
22 Exploration analysis on generating cell morphology images with the L1000 gene expression profile on those perturbations without corresponding ground-truth cell morphology images. 32
23 The drug structures of BML-259, RG-14620, and BRD-A72066420. 33
24 The UMAP projection for different data, including drug embeddings, gene expression and DeepProfiler embeddings. 33
25 Analysis of the potential impact of the quality of the DMSO control morphology images on the performance of the I2I mode of MorphDiff as well as practical tips. 34
26 Statistics showing the Wasserstein Distance between the DeepProfiler embeddings of the training dataset and the MOA/target set in the CDRP dataset. Source data are provided as a Source Data file. 35
27 Evaluating the use cases and applicability of MorphDiff. 36
28 Correlation between the Wasserstein Distance of the training and ground-truth OOD data and the normalized metrics at Target and MOA levels. 37
29 Correlation between the Wasserstein Distance of training gene count and inference OOD L1000 gene count and the normalized metrics at Target and MOA levels. 38
30 Comparison of the performance of MorphDiff methods and IMPA method on the generalization capabilities with respect to the distance of drug embedding / gene count. 39

A Supplementary Notes

A.1 Baseline Methods

We used seven common baselines in cell morphology generation and image translation covering diverse generative network paradigms to comprehensively benchmark the performance of our method. Unless otherwise stated, we used the default hyperparameters of these baseline methods.

MorphNet MorphNet [1] is a computational approach that can infer images of a cell's morphology from its gene expression. MorphNet contains two main components, the Variational Autoencoder (VAE) and Generative Adversarial Network (GAN). In the first training stage, the VAE is trained on gene expression with ELBO loss to generate low-dimensional gene expression embeddings [2]. In the second training stage, the GAN network adapted from StyleGAN2 is used to generate cell images conditioned on low-dimensional gene expression embeddings. In the inference stage, the gene expression is first encoded with the VAE encoder and then the cell images are generated through the GAN network.

IMPA IMPA (IMage Perturbation Autoencoder) [3] is proposed to predict the cellular morphological effects of various kinds of perturbations using untreated cells as input. The model architecture is based on StarGANv2 [4]. Concretely, the model takes a control cell image as input, and the image is encoded into a dense representation. One perturbation embedding is sampled and concatenated with a randomly distributed vector. The concatenated embeddings are named style embeddings and then fed into a perturbation encoder. The encoded embedding will be used to condition every layer in the decoder. A perturbation discriminator will be employed to classify the specific perturbation from the decoder output. Meanwhile, a style encoder is trained to replicate the style vector from the decoder output image.

StarGANv1 StarGANv1 [5] performs conditional image-to-image translation across multiple domains using a single adversarial network. The objective is to learn a single generator for mapping across several different domains. During the training process, the discriminator is trained to minimize the classification error between ground truth and generated data. The generator aims to fool the discriminator with generated data on ground-truth/generated classification and maximize the classification accuracy across domains.

DRIT++ DRIT++ (Disentangled Representation for Image-to-Image Translation) [6] is a powerful tool in domain adaptation and image translation. It encodes images into a shared content space and domain-specific attribute space. The content encoders are trained to produce content embeddings that are not distinguishable by the attribute discriminator. The cross-cycle consistency loss is adapted to exploit the disentangled content and attribute representations for cyclic reconstruction.

DMIT DMIT (Disentanglement for Multi-mapping Image-to-Image Translation) [7] is a GAN framework developed for multi-condition image translation. The DMIT learning process can be separated into the disentanglement path and the translation path. The input images will first be disentangled into the latent representation by an encoder-decoder architecture with conditional adversarial training. The generator will then learn multi-mappings across different domains by randomly performing cross-domain translation.

VQGAN VQGAN (Vector Quantised Generative Adversarial Network) [8] combines the inductive bias of CNNs with the expressive power of transformers to model and synthesize high-resolution images. It employs CNNs to learn a context-rich vocabulary of image components and uses transformers to efficiently model their composition in high-resolution images. VQGAN is well suited for conditional synthesis tasks, accommodating both non-spatial information, like object classes, and spatial information.

MDTv2 MDTv2 (Masked Diffusion Transformer) [9] introduces a masked latent modeling scheme to learn contextual relationships among semantic parts of an image. During training, MDTv2 masks certain tokens in the latent space. An asymmetric diffusion transformer predicts these masked tokens from the unmasked ones with the diffusion generation process. MDTv2 can reconstruct complete image information from incomplete inputs, learning relationships among image tokens. A further improved MDT with a more efficient macro network structure and training strategy is named MDTv2.

A.2 Criteria for choosing the images for visualization

As the models can generate multiple images for a given perturbation, we established the following criteria for selecting images for visualization. For each row of images, we selected the generated image from each method that had the highest structural similarity (SSIM) [10] to the ground-truth image. This approach prevents selective bias and ensures fair visual comparison across methods.

A.3 Analysis of the impact of the quality of the control morphology images on I2I mode performance as well as practical tips

Furthermore, we conducted preliminary experiments to explore how specific properties of control morphology images affect MorphDiff's I2I mode performance during inference. Like most large-scale generative models, diffusion architectures operate as complex systems whose internal mechanisms are not fully transparent. Rather than attempting theoretical analysis, our approach will focus on empirical observations to identify potential factors influencing performance variation. This practical investigation aligns with the current state of machine learning research, where complete interpretability of such models remains an ongoing challenge in the field.

Concretely, we investigate the impact of kurtosis properties [11] of the reference DMSO control morphology image set on the performance of the sampled output images produced by the I2I mode of MorphDiff. Kurtosis, which quantifies the degree of "tailedness" in a distribution, is potentially relevant in this context because high kurtosis suggests a higher likelihood of extreme outliers in the control morphology image data. We hypothesize that the presence of outliers in the control reference morphology images might influence generative model performance, suggesting kurtosis could serve as a potential metric for assessing dataset characteristics. We sampled 100 sets of control morphology images from the whole pool of control DMSO morphology images and calculated the kurtosis of DeepProfiler embeddings for each set in a multivariate manner [11]. We then sampled MorphDiff with the I2I mode using each set of control DMSO morphology images and assessed the generative performance. Through this approach, we explored the possible relationship between the kurtosis of the reference control DMSO morphology image sets and their resulting generative performance.

We assume that higher kurtosis is associated with a higher likelihood of extreme values, which may contribute to degraded model performance in the generated outputs. The results on the JUMP OOD dataset and the CDRP OOD dataset are shown in Supplementary Figure 25a and b. We observed that the kurtosis of the reference DMSO morphology image set (x-axis) has a negative correlation with the performance metrics of the generated output (y-axis) on both datasets. This supports Our analysis revealed that the kurtosis of the reference DMSO images can be one of the potential factors causing these discrepancies.

We also provided practical tips for applying the I2I mode of the MorphDiff model to enhance the method's robustness. As revealed in Supplementary Figure 25(a-b), higher kurtosis in control reference morphology images suggests a greater propensity for outliers, which may negatively impact generative model performance. Therefore, filtering outlier samples from the control reference cell morphology set could potentially improve the robustness of results. To provide quantitative validation of this approach, we conducted the following analysis as illustrated in Supplementary Figure 25c: for a given control reference cell morphology set, we used Mahalanobis distance (the distance used to compute kurtosis [11]) to measure the distance from distribution center to each sample, where closer distance indicates samples less likely to be outliers [12]. We then compared the performance between two scenarios: (1) retaining only the closest $X\%$ of samples to the center (those least likely to be outliers) and (2) retaining only the furthest $X\%$ of samples from the center (those most likely to be outliers). As shown in Supplementary Figure 25d, on the JUMP OOD dataset, using the closest $X\%$ of samples led to performance improvements compared to using the furthest $X\%$ of samples. The improvements were particularly notable in FID(-1) and Inception Scores, demonstrating that filtering outliers based on Mahalanobis distance from the center before applying MorphDiff (I2I) can effectively enhance the robustness of the sampling results as shown in Supplementary Figure 25e.

A.4 Analyzing the applicability of MorphDiff on OOD MOAs and targets

Prediction models may perform better on data that bear greater similarity to the training data, which is a fundamental consideration in machine learning that merits careful attention when evaluating predictive capabilities. We applied this principle by inferring our model on unseen data. First, we analyzed the distance between the training dataset and the MOA/Target dataset in the CDRP dataset, with results shown in Supplementary Figure 26.

Then, we used a straightforward approach to quantify the generalization capability of prediction models when handling out-of-domain test datasets belonging to different drugs, MOAs, and targets. Specifically, we calculated

the Wasserstein Distance between the DeepProfiler embeddings of the CDRP Training set and the ground-truth CDRP Target_MOA dataset. We examined how performance on different metrics change with the Wasserstein Distance between the training and testing datasets at both target and MOA levels. The results are shown in Supplementary Figure 27a and Supplementary Figure 28. As demonstrated, we found that all metrics exhibit a consistent negative correlation with respect to the distance with the CDRP Training set. Overall, we observe that model performance is affected by the distance between training and testing datasets, with degraded performance on more remote testing datasets. On the MOA level, our model makes the best predictions on HMGCR inhibitor, PPAR receptor agonist and EGFR inhibitor. On the target level, our model makes the best predictions on *PPARG*, *EGFR* and *CDK1*.

Having found that the distance between ground-truth cell morphology and training cell morphology can impact model performance, we next sought to provide practical user guidelines. In real-world applications, users will input perturbed gene counts directly into the MorphDiff model and utilize the predicted cell morphology responses without knowing the ground-truth cell morphology distribution. Therefore, we quantified whether the Wasserstein Distance between the training gene counts and the inference gene counts affects model performance. As shown in Supplementary Figure 27b and Supplementary Figure 29, there is indeed a negative correlation between this distance and performance metrics. This indicates that models experience degraded and less reliable performance when inferring cell morphology using gene count distributions that are distant from those in the training set. We also compared the performance of MorphDiff with IMPA in Supplementary Figure 27c and Supplementary Figure 30. Specifically, we divided the drug MOAs in the out-of-distribution (OOD) set into five equal-sized bins based on their distance from the training dataset, calculated using drug embeddings (the input of IMPA) and gene counts (the input of MorphDiff). We found that under both settings, regardless of whether the test input conditions were closer to or further from the training distribution, MorphDiff consistently outperforms the baseline method in most cases, highlighting MorphDiff's superior generalizability when predicting OOD perturbations.

In Supplementary Figure 27d, we present an illustrative guideline for method users. When users want to apply the model to infer cell morphological responses from new L1000 gene expression profiles in their applications, we suggest first checking the similarity between the new L1000 profile and the L1000 database used in MorphDiff training. Higher similarity indicates that results are more likely to be reliable; conversely, lower similarity suggests that users should exercise caution, as predicting distant perturbations in out-of-distribution (OOD) scenarios remains challenging for all tools, though MorphDiff demonstrates superior performance compared to other tools in such cases. This phenomenon has also been discussed in previous works related to predicting morphological perturbation response and transcriptional response. For example, in IMPA [3], the authors explicitly acknowledged that "its predictive ability and performance for perturbations that are very different from those in the training set may decrease." Similarly, CellOT [13], which focuses on predicting transcriptional response, stated that "The ability to make o.o.d. predictions, however, is only feasible if (1) similar samples have been observed in the unperturbed setting and (2) the training set contains cases that are similar not only in their unperturbed state but also their perturbation response." These observations reinforce our position that predicting distant OOD perturbations remains challenging for current methods. Addressing this challenge will require both the curation of large-scale, high-quality, comprehensive datasets and the development of novel learning paradigms.

B Supplementary Tables

Supplementary Table 1: The CellProfiler channel name refers to the name assigned by the CellProfiler to each channel. This table describes the cellular component corresponding to the CellProfiler channel name and the dye used for imaging.

Dye	Organelle or cellular component	CellProfiler
Hoechst 33342	Nucleus	DNA
Concanavalin A/Alexa Fluor 488 conjugate	Endoplasmic reticulum	ER
SYTO 14 green fluorescent nucleic acid stain	Nucleoli, cytoplasmic RNA	RNA
Phalloidin/Alexa Fluor 594 conjugate, wheat germ agglutinin (WGA)/Alexa Fluor 594 conjugate	F-actin cytoskeleton, Golgi, plasma membrane	AGP
MitoTracker Deep Red	Mitochondria	Mito

Supplementary Table 2: Comparison of MorphDiff with other related tools. G2I (Gene-to-Image) means whether this tool supports perturbed gene expression/gene embeddings/drug embeddings to perturbed cell morphology generation. I2I (Image-to-Image) means whether this tool supports control cell morphology to perturbed cell morphology generation.

Model	G2I	I2I
MorphDiff	Y	Y
StarGANv1	N	Y
IMPA	N	Y
DMIT	N	Y
DRIT++	N	Y
MDTv2	Y	N
VQGAN	Y	N
MorphNet	Y	N

Supplementary Table 3: Detailed results of the Maximum Mean Discrepancy (MMD) test on the CDRP dataset (Repeat 10 times). It is a two-sided test and adjustments for multiple comparisons are not applicable.

Dataset	Rounds	Statistics	p-value	Dataset	Rounds	Statistics	p-value
ID set		-2.62E-05	0.714	ID set		3.92E-05	0.164
OOD set	0	1.88E-03	0.001	OOD set	5	2.05E-03	0.001
Target_MOA set		7.92E-02	0.001	Target_MOA set		7.79E-02	0.001
ID set		5.28E-05	0.103	ID set		-4.06E-05	0.851
OOD set	1	1.94E-03	0.001	OOD set	6	2.26E-03	0.001
Target_MOA set		7.85E-02	0.001	Target_MOA set		8.18E-02	0.001
ID set		-1.15E-05	0.550	ID set		2.32E-05	0.251
OOD set	2	1.99E-03	0.001	OOD set	7	2.44E-03	0.001
Target_MOA set		7.86E-02	0.001	Target_MOA set		8.12E-02	0.001
ID set		-2.58E-05	0.713	ID set		-9.71E-06	0.509
OOD set	3	1.99E-03	0.001	OOD set	8	1.54E-03	0.001
Target_MOA set		7.83E-02	0.001	Target_MOA set		7.75E-02	0.001
ID set		-4.59E-06	0.463	ID set		-3.44E-05	0.803
OOD set	4	1.98E-03	0.001	OOD set	9	1.88E-03	0.001
Target_MOA set		7.78E-02	0.001	Target_MOA set		7.99E-02	0.001

Supplementary Table 4: Detailed results of the Energy Distance test on the CDRP dataset (Repeat 10 times). It is a two-sided test and adjustments for multiple comparisons are not applicable.

Dataset	Rounds	Statistics	p-value	Dataset	Rounds	Statistics	p-value
ID set		9.815	0.726	ID set		12.412	0.163
OOD set	0	82.562	0.001	OOD set	5	88.560	0.001
Target_MOA set		2808.427	0.001	Target_MOA set		2761.942	0.001
ID set		12.983	0.114	ID set		9.501	0.829
OOD set	1	83.840	0.001	OOD set	6	96.208	0.001
Target_MOA set		2773.373	0.001	Target_MOA set		2895.760	0.001
ID set		10.608	0.553	ID set		11.916	0.253
OOD set	2	86.011	0.001	OOD set	7	102.112	0.001
Target_MOA set		2776.654	0.001	Target_MOA set		2867.429	0.001
ID set		10.218	0.608	ID set		10.627	0.518
OOD set	3	85.052	0.001	OOD set	8	69.067	0.001
Target_MOA set		2773.926	0.001	Target_MOA set		2742.465	0.001
ID set		10.790	0.481	ID set		9.620	0.817
OOD set	4	84.535	0.001	OOD set	9	81.002	0.001
Target_MOA set		2750.999	0.001	Target_MOA set		2828.478	0.001

Supplementary Table 5: Detailed results of the Maximum Mean Discrepancy (MMD) test on the JUMP dataset (Repeat 10 times). It is a two-sided test and adjustments for multiple comparisons are not applicable.

Dataset	Rounds	Statistics	p-value	Dataset	Rounds	Statistics	p-value
ID set	0	1.61E-05	0.308	ID set	5	-2.25E-06	0.425
OOD set		1.14E-02	0.001	OOD set		9.91E-03	0.001
ID set	1	-1.62E-05	0.568	ID set	6	-4.22E-05	0.816
OOD set		9.77E-03	0.001	OOD set		1.01E-02	0.001
ID set	2	-5.82E-05	0.941	ID set	7	4.48E-05	0.154
OOD set		1.06E-02	0.001	OOD set		1.07E-02	0.001
ID set	3	5.34E-05	0.129	ID set	8	-2.28E-05	0.638
OOD set		1.08E-02	0.001	OOD set		1.07E-02	0.001
ID set	4	1.75E-05	0.300	ID set	9	-8.38E-06	0.507
OOD set		1.03E-02	0.001	OOD set		1.03E-02	0.001

Supplementary Table 6: Detailed results of the Energy Distance test on the JUMP dataset (Repeat 10 times). It is a two-sided test and adjustments for multiple comparisons are not applicable.

Dataset	Rounds	Statistics	p-value	Dataset	Rounds	Statistics	p-value
ID set	0	9.815	0.726	ID set	5	12.412	0.163
OOD set		82.562	0.001	OOD set		88.560	0.001
ID set	1	12.983	0.114	ID set	6	9.501	0.829
OOD set		83.840	0.001	OOD set		96.208	0.001
ID set	2	10.608	0.553	ID set	7	11.916	0.253
OOD set		86.011	0.001	OOD set		102.112	0.001
ID set	3	10.218	0.608	ID set	8	10.627	0.518
OOD set		85.052	0.001	OOD set		69.067	0.001
ID set	4	10.790	0.481	ID set	9	9.620	0.817
OOD set		84.535	0.001	OOD set		81.002	0.001

Supplementary Table 7: Detailed results of the Maximum Mean Discrepancy (MMD) on the LINCS Target leave-one-out set (Repeat 10 times). It is a two-sided test and adjustments for multiple comparisons are not applicable.

Target	Rounds	Statistics	p-value	Target	Rounds	Statistics	p-value
CTSK		0.021	0.001	*CTSK*		0.033	0.001
ERBB2		0.021	0.001	*ERBB2*		0.035	0.001
F10		0.021	0.001	*F10*		0.034	0.001
MELK		0.022	0.001	*MELK*		0.035	0.001
MLNR	0	0.021	0.001	*MLNR*	5	0.034	0.001
PREP		0.021	0.001	*PREP*		0.035	0.001
PTGS2		0.022	0.001	*PTGS2*		0.035	0.001
SRGAP1		0.020	0.001	*SRGAP1*		0.034	0.001
TOP2A		0.021	0.001	*TOP2A*		0.033	0.001
TRPA1		0.020	0.001	*TRPA1*		0.035	0.001
CTSK		0.009	0.001	*CTSK*		0.004	0.001
ERBB2		0.010	0.001	*ERBB2*		0.004	0.001
F10		0.010	0.001	*F10*		0.004	0.001
MELK		0.011	0.001	*MELK*		0.004	0.001
MLNR	1	0.010	0.001	*MLNR*	6	0.004	0.001
PREP		0.010	0.001	*PREP*		0.004	0.001
PTGS2		0.010	0.001	*PTGS2*		0.004	0.001
SRGAP1		0.010	0.001	*SRGAP1*		0.004	0.001
TOP2A		0.010	0.001	*TOP2A*		0.004	0.001
TRPA1		0.010	0.001	*TRPA1*		0.004	0.001
CTSK		0.016	0.001	*CTSK*		0.006	0.001
ERBB2		0.018	0.001	*ERBB2*		0.006	0.001
F10		0.017	0.001	*F10*		0.006	0.001
MELK		0.018	0.001	*MELK*		0.006	0.001
MLNR	2	0.017	0.001	*MLNR*	7	0.006	0.001
PREP		0.018	0.001	*PREP*		0.006	0.001
PTGS2		0.017	0.001	*PTGS2*		0.006	0.001
SRGAP1		0.018	0.001	*SRGAP1*		0.006	0.001
TOP2A		0.017	0.001	*TOP2A*		0.006	0.001
TRPA1		0.017	0.001	*TRPA1*		0.006	0.001
CTSK		0.004	0.001	*CTSK*		0.008	0.001
ERBB2		0.004	0.001	*ERBB2*		0.009	0.001
F10		0.004	0.001	*F10*		0.008	0.001
MELK		0.004	0.001	*MELK*		0.008	0.001
MLNR	3	0.005	0.001	*MLNR*	8	0.008	0.001
PREP		0.004	0.001	*PREP*		0.008	0.001
PTGS2		0.005	0.001	*PTGS2*		0.009	0.001
SRGAP1		0.004	0.001	*SRGAP1*		0.009	0.001
TOP2A		0.004	0.001	*TOP2A*		0.009	0.001
TRPA1		0.004	0.001	*TRPA1*		0.009	0.001
CTSK		0.005	0.001	*CTSK*		0.027	0.001
ERBB2		0.004	0.001	*ERBB2*		0.029	0.001
F10		0.005	0.001	*F10*		0.029	0.001
MELK		0.005	0.001	*MELK*		0.029	0.001
MLNR	4	0.005	0.001	*MLNR*	9	0.028	0.001
PREP		0.005	0.001	*PREP*		0.027	0.001
PTGS2		0.005	0.001	*PTGS2*		0.027	0.001
SRGAP1		0.004	0.001	*SRGAP1*		0.027	0.001
TOP2A		0.005	0.001	*TOP2A*		0.027	0.001
TRPA1		0.004	0.001	*TRPA1*		0.029	0.001

Supplementary Table 8: Detailed results of the Energy Distance Test on the LINCS Target leave-one-out set (Repeat 10 times). It is a two-sided test and adjustments for multiple comparisons are not applicable.

Target	Rounds	Statistics	p-value	Target	Rounds	Statistics	p-value
CTSK	0	774.683	0.001	*CTSK*	5	1271.801	0.001
ERBB2		791.469	0.001	*ERBB2*		1322.075	0.001
F10		799.256	0.001	*F10*		1301.862	0.001
MELK		834.459	0.001	*MELK*		1346.745	0.001
MLNR		790.459	0.001	*MLNR*		1290.705	0.001
PREP		779.179	0.001	*PREP*		1328.801	0.001
PTGS2		821.662	0.001	*PTGS2*		1318.103	0.001
SRGAP1		765.466	0.001	*SRGAP1*		1304.944	0.001
TOP2A		779.625	0.001	*TOP2A*		1275.148	0.001
TRPA1		763.520	0.001	*TRPA1*		1325.110	0.001
CTSK	1	370.440	0.001	*CTSK*	6	164.550	0.001
ERBB2		407.110	0.001	*ERBB2*		168.736	0.001
F10		379.061	0.001	*F10*		169.201	0.001
MELK		419.972	0.001	*MELK*		151.882	0.001
MLNR		394.402	0.001	*MLNR*		173.193	0.001
PREP		379.386	0.001	*PREP*		176.399	0.001
PTGS2		404.070	0.001	*PTGS2*		152.814	0.001
SRGAP1		380.579	0.001	*SRGAP1*		176.289	0.001
TOP2A		377.674	0.001	*TOP2A*		163.058	0.001
TRPA1		400.459	0.001	*TRPA1*		169.827	0.001
CTSK	2	630.308	0.001	*CTSK*	7	266.772	0.001
ERBB2		670.262	0.001	*ERBB2*		246.107	0.001
F10		640.987	0.001	*F10*		266.530	0.001
MELK		674.095	0.001	*MELK*		255.561	0.001
MLNR		639.816	0.001	*MLNR*		248.198	0.001
PREP		674.203	0.001	*PREP*		242.636	0.001
PTGS2		664.330	0.001	*PTGS2*		249.065	0.001
SRGAP1		684.513	0.001	*SRGAP1*		245.500	0.001
TOP2A		660.435	0.001	*TOP2A*		247.638	0.001
TRPA1		657.089	0.001	*TRPA1*		246.702	0.001
CTSK	3	183.508	0.001	*CTSK*	8	312.442	0.001
ERBB2		177.683	0.001	*ERBB2*		339.042	0.001
F10		180.307	0.001	*F10*		315.281	0.001
MELK		182.482	0.001	*MELK*		309.171	0.001
MLNR		192.208	0.001	*MLNR*		328.685	0.001
PREP		166.677	0.001	*PREP*		323.465	0.001
PTGS2		186.878	0.001	*PTGS2*		348.552	0.001
SRGAP1		162.949	0.001	*SRGAP1*		338.639	0.001
TOP2A		173.872	0.001	*TOP2A*		340.584	0.001
TRPA1		173.300	0.001	*TRPA1*		331.113	0.001
CTSK	4	191.854	0.001	*CTSK*	9	1069.068	0.001
ERBB2		181.950	0.001	*ERBB2*		1132.253	0.001
F10		215.084	0.001	*F10*		1119.731	0.001
MELK		198.643	0.001	*MELK*		1109.266	0.001
MLNR		205.765	0.001	*MLNR*		1087.651	0.001
PREP		194.802	0.001	*PREP*		1069.201	0.001
PTGS2		197.199	0.001	*PTGS2*		1053.327	0.001
SRGAP1		183.812	0.001	*SRGAP1*		1047.547	0.001
TOP2A		187.783	0.001	*TOP2A*		1072.184	0.001
TRPA1		179.384	0.001	*TRPA1*		1129.584	0.001

Supplementary Table 9: Detailed results of the Maximum Mean Discrepancy on the LINCS MOA leave-one-out set (Repeat 10 times). It is a two-sided test and adjustments for multiple comparisons are not applicable.

MOA	Rounds	Statistics	p-value	MOA	Rounds	Statistics	p-value
EGFR inhibitor	0	0.004	0.001	EGFR inhibitor	5	0.001	0.001
HMGCR inhibitor		0.004	0.001	HMGCR inhibitor		0.001	0.001
NFkB pathway inhibitor		0.004	0.001	NFkB pathway inhibitor		0.001	0.001
adrenergic receptor agonist		0.004	0.001	adrenergic receptor agonist		0.001	0.001
adrenergic receptor inhibitor		0.003	0.001	adrenergic receptor inhibitor		0.001	0.001
calcium channel blocker		0.004	0.001	calcium channel blocker		0.001	0.001
dopamine receptor agonist		0.004	0.001	dopamine receptor agonist		0.001	0.001
dopamine receptor inhibitor		0.004	0.001	dopamine receptor inhibitor		0.001	0.001
phosphodiesterase inhibitor		0.004	0.001	phosphodiesterase inhibitor		0.001	0.001
serotonin receptor antagonist		0.004	0.001	serotonin receptor antagonist		0.001	0.001
EGFR inhibitor	1	0.010	0.001	EGFR inhibitor	6	0.018	0.001
HMGCR inhibitor		0.009	0.001	HMGCR inhibitor		0.018	0.001
NFkB pathway inhibitor		0.010	0.001	NFkB pathway inhibitor		0.018	0.001
adrenergic receptor agonist		0.009	0.001	adrenergic receptor agonist		0.018	0.001
adrenergic receptor inhibitor		0.010	0.001	adrenergic receptor inhibitor		0.019	0.001
calcium channel blocker		0.009	0.001	calcium channel blocker		0.018	0.001
dopamine receptor agonist		0.009	0.001	dopamine receptor agonist		0.018	0.001
dopamine receptor inhibitor		0.010	0.001	dopamine receptor inhibitor		0.017	0.001
phosphodiesterase inhibitor		0.009	0.001	phosphodiesterase inhibitor		0.018	0.001
serotonin receptor antagonist		0.010	0.001	serotonin receptor antagonist		0.019	0.001
EGFR inhibitor	2	0.002	0.001	EGFR inhibitor	7	0.002	0.001
HMGCR inhibitor		0.003	0.001	HMGCR inhibitor		0.003	0.001
NFkB pathway inhibitor		0.003	0.001	NFkB pathway inhibitor		0.002	0.001
adrenergic receptor agonist		0.002	0.001	adrenergic receptor agonist		0.002	0.001
adrenergic receptor inhibitor		0.003	0.001	adrenergic receptor inhibitor		0.003	0.001
calcium channel blocker		0.003	0.001	calcium channel blocker		0.003	0.001
dopamine receptor agonist		0.003	0.001	dopamine receptor agonist		0.002	0.001
dopamine receptor inhibitor		0.002	0.001	dopamine receptor inhibitor		0.003	0.001
phosphodiesterase inhibitor		0.003	0.001	phosphodiesterase inhibitor		0.003	0.001
serotonin receptor antagonist		0.002	0.001	serotonin receptor antagonist		0.002	0.001
EGFR inhibitor	3	0.012	0.001	EGFR inhibitor	8	0.002	0.001
HMGCR inhibitor		0.012	0.001	HMGCR inhibitor		0.002	0.001
NFkB pathway inhibitor		0.012	0.001	NFkB pathway inhibitor		0.002	0.001
adrenergic receptor agonist		0.013	0.001	adrenergic receptor agonist		0.002	0.001
adrenergic receptor inhibitor		0.012	0.001	adrenergic receptor inhibitor		0.002	0.001
calcium channel blocker		0.011	0.001	calcium channel blocker		0.002	0.001
dopamine receptor agonist		0.012	0.001	dopamine receptor agonist		0.002	0.001
dopamine receptor inhibitor		0.012	0.001	dopamine receptor inhibitor		0.002	0.001
phosphodiesterase inhibitor		0.012	0.001	phosphodiesterase inhibitor		0.002	0.001
serotonin receptor antagonist		0.012	0.001	serotonin receptor antagonist		0.002	0.001
EGFR inhibitor	4	0.016	0.001	EGFR inhibitor	9	0.005	0.001
HMGCR inhibitor		0.016	0.001	HMGCR inhibitor		0.005	0.001
NFkB pathway inhibitor		0.017	0.001	NFkB pathway inhibitor		0.006	0.001
adrenergic receptor agonist		0.015	0.001	adrenergic receptor agonist		0.005	0.001
adrenergic receptor inhibitor		0.015	0.001	adrenergic receptor inhibitor		0.005	0.001
calcium channel blocker		0.016	0.001	calcium channel blocker		0.006	0.001
dopamine receptor agonist		0.016	0.001	dopamine receptor agonist		0.005	0.001
dopamine receptor inhibitor		0.016	0.001	dopamine receptor inhibitor		0.005	0.001
phosphodiesterase inhibitor		0.016	0.001	phosphodiesterase inhibitor		0.005	0.001
serotonin receptor antagonist		0.016	0.001	serotonin receptor antagonist		0.006	0.001

Supplementary Table 10: Detailed results of the Energy Distance Test on the LINCS MOA leave-one-out set (Repeat 10 times). It is a two-sided test and adjustments for multiple comparisons are not applicable.

MOA	Rounds	Statistics	p-value	MOA	Rounds	Statistics	p-value
EGFR inhibitor		162.594	0.001	EGFR inhibitor		35.816	0.001
HMGCR inhibitor		154.919	0.001	HMGCR inhibitor		37.866	0.001
NFkB pathway inhibitor		147.968	0.001	NFkB pathway inhibitor		52.126	0.001
adrenergic receptor agonist		159.153	0.001	adrenergic receptor agonist		53.919	0.001
adrenergic receptor inhibitor	0	138.315	0.001	adrenergic receptor inhibitor	5	51.239	0.001
calcium channel blocker		161.838	0.001	calcium channel blocker		40.528	0.001
dopamine receptor agonist		146.804	0.001	dopamine receptor agonist		45.129	0.001
dopamine receptor inhibitor		147.282	0.001	dopamine receptor inhibitor		46.806	0.001
phosphodiesterase inhibitor		161.655	0.001	phosphodiesterase inhibitor		45.250	0.001
serotonin receptor antagonist		145.992	0.001	serotonin receptor antagonist		42.078	0.001
EGFR inhibitor		364.421	0.001	EGFR inhibitor		679.458	0.001
HMGCR inhibitor		362.582	0.001	HMGCR inhibitor		687.616	0.001
NFkB pathway inhibitor		366.814	0.001	NFkB pathway inhibitor		702.750	0.001
adrenergic receptor agonist		363.883	0.001	adrenergic receptor agonist		683.194	0.001
adrenergic receptor inhibitor	1	383.216	0.001	adrenergic receptor inhibitor	6	716.722	0.001
calcium channel blocker		361.565	0.001	calcium channel blocker		703.348	0.001
dopamine receptor agonist		350.055	0.001	dopamine receptor agonist		698.971	0.001
dopamine receptor inhibitor		371.723	0.001	dopamine receptor inhibitor		662.825	0.001
phosphodiesterase inhibitor		349.038	0.001	phosphodiesterase inhibitor		674.459	0.001
serotonin receptor antagonist		379.420	0.001	serotonin receptor antagonist		708.494	0.001
EGFR inhibitor		106.280	0.001	EGFR inhibitor		98.334	0.001
HMGCR inhibitor		119.521	0.001	HMGCR inhibitor		113.771	0.001
NFkB pathway inhibitor		113.592	0.001	NFkB pathway inhibitor		92.020	0.001
adrenergic receptor agonist		109.492	0.001	adrenergic receptor agonist		105.139	0.001
adrenergic receptor inhibitor	2	119.698	0.001	adrenergic receptor inhibitor	7	110.331	0.001
calcium channel blocker		123.264	0.001	calcium channel blocker		112.086	0.001
dopamine receptor agonist		130.497	0.001	dopamine receptor agonist		105.902	0.001
dopamine receptor inhibitor		106.897	0.001	dopamine receptor inhibitor		110.970	0.001
phosphodiesterase inhibitor		116.367	0.001	phosphodiesterase inhibitor		107.899	0.001
serotonin receptor antagonist		103.285	0.001	serotonin receptor antagonist		97.183	0.001
EGFR inhibitor		467.787	0.001	EGFR inhibitor		76.362	0.001
HMGCR inhibitor		470.290	0.001	HMGCR inhibitor		86.119	0.001
NFkB pathway inhibitor		460.696	0.001	NFkB pathway inhibitor		77.671	0.001
adrenergic receptor agonist		491.312	0.001	adrenergic receptor agonist		74.922	0.001
adrenergic receptor inhibitor	3	466.398	0.001	adrenergic receptor inhibitor	8	81.949	0.001
calcium channel blocker		448.481	0.001	calcium channel blocker		74.335	0.001
dopamine receptor agonist		479.226	0.001	dopamine receptor agonist		77.346	0.001
dopamine receptor inhibitor		476.312	0.001	dopamine receptor inhibitor		78.339	0.001
phosphodiesterase inhibitor		462.820	0.001	phosphodiesterase inhibitor		82.342	0.001
serotonin receptor antagonist		464.876	0.001	serotonin receptor antagonist		77.242	0.001
EGFR inhibitor		615.468	0.001	EGFR inhibitor		199.137	0.001
HMGCR inhibitor		627.009	0.001	HMGCR inhibitor		196.891	0.001
NFkB pathway inhibitor		645.583	0.001	NFkB pathway inhibitor		221.902	0.001
adrenergic receptor agonist		592.643	0.001	adrenergic receptor agonist		208.717	0.001
adrenergic receptor inhibitor	4	592.216	0.001	adrenergic receptor inhibitor	9	200.890	0.001
calcium channel blocker		625.183	0.001	calcium channel blocker		220.990	0.001
dopamine receptor agonist		631.829	0.001	dopamine receptor agonist		201.646	0.001
dopamine receptor inhibitor		618.807	0.001	dopamine receptor inhibitor		192.499	0.001
phosphodiesterase inhibitor		616.610	0.001	phosphodiesterase inhibitor		203.380	0.001
serotonin receptor antagonist		611.908	0.001	serotonin receptor antagonist		226.804	0.001

C Supplementary Figures

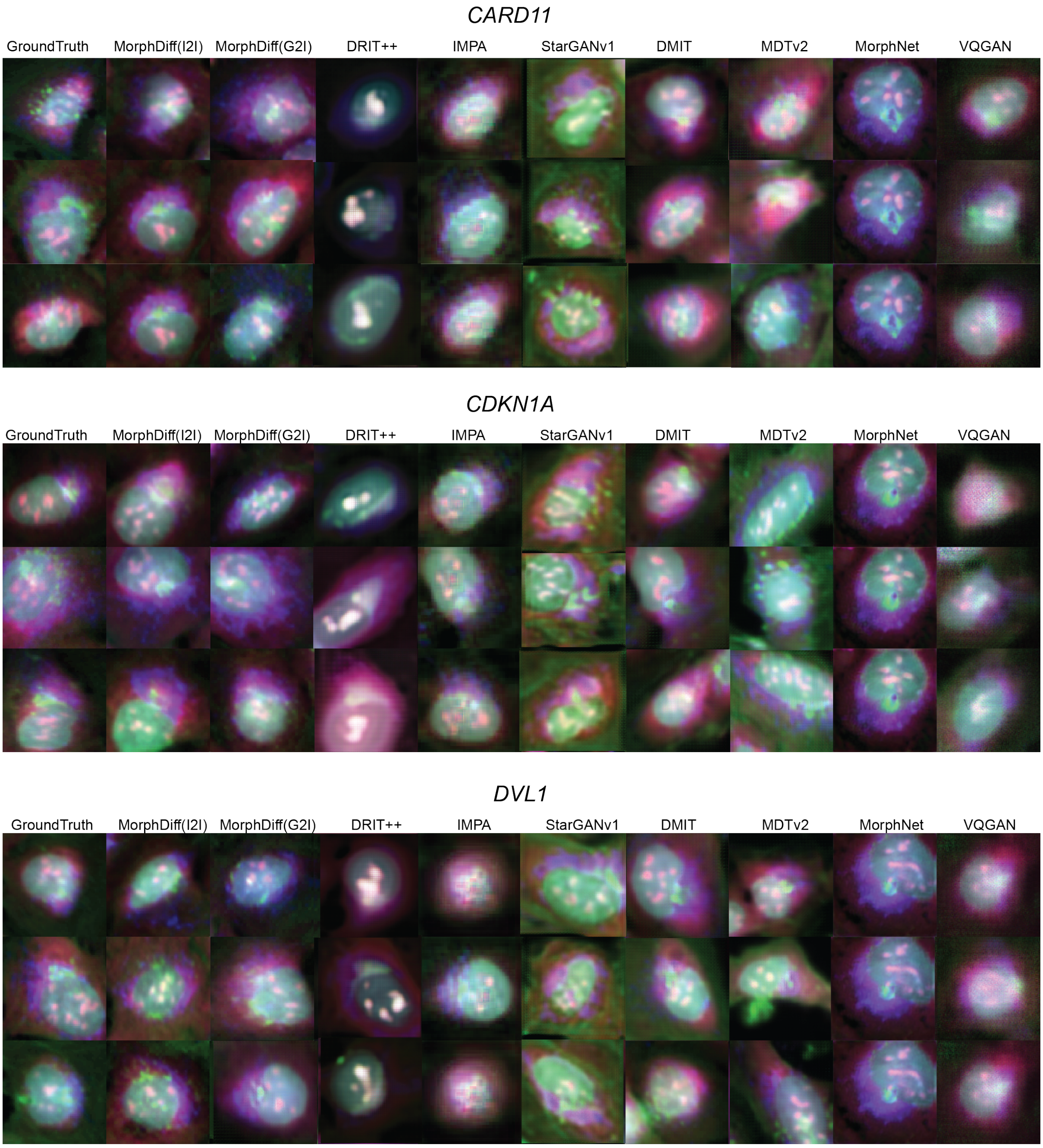

Supplementary Figure 1: Visualization of the generated images from each baseline method as well as the ground-truth from the JUMP dataset. We randomly chose three ground-truth images for each genetic perturbation, and select generated images of baseline by SSIM[10] for visualization. This figure visualizes the results for *CARD11*, *CDKN11* and *DVL1* genetic perturbations. MorphDiff (both modes) stands out as the top performer in terms of visual quality and detail across all perturbations. DMIT, IMPA and MDTv2 offer decent performance but generally fall short in detail and clarity. MorphNet generates reasonable images with some detail, but lacks diversity, and the detail is inconsistent with the ground truth. DRIT++, StarGANv1 and VQGAN exhibit varying levels of quality, often resulting in less clear images. The visual quality can be inconsistent with ground truth, with some images appearing blurry. The scale bar is 20 μm.

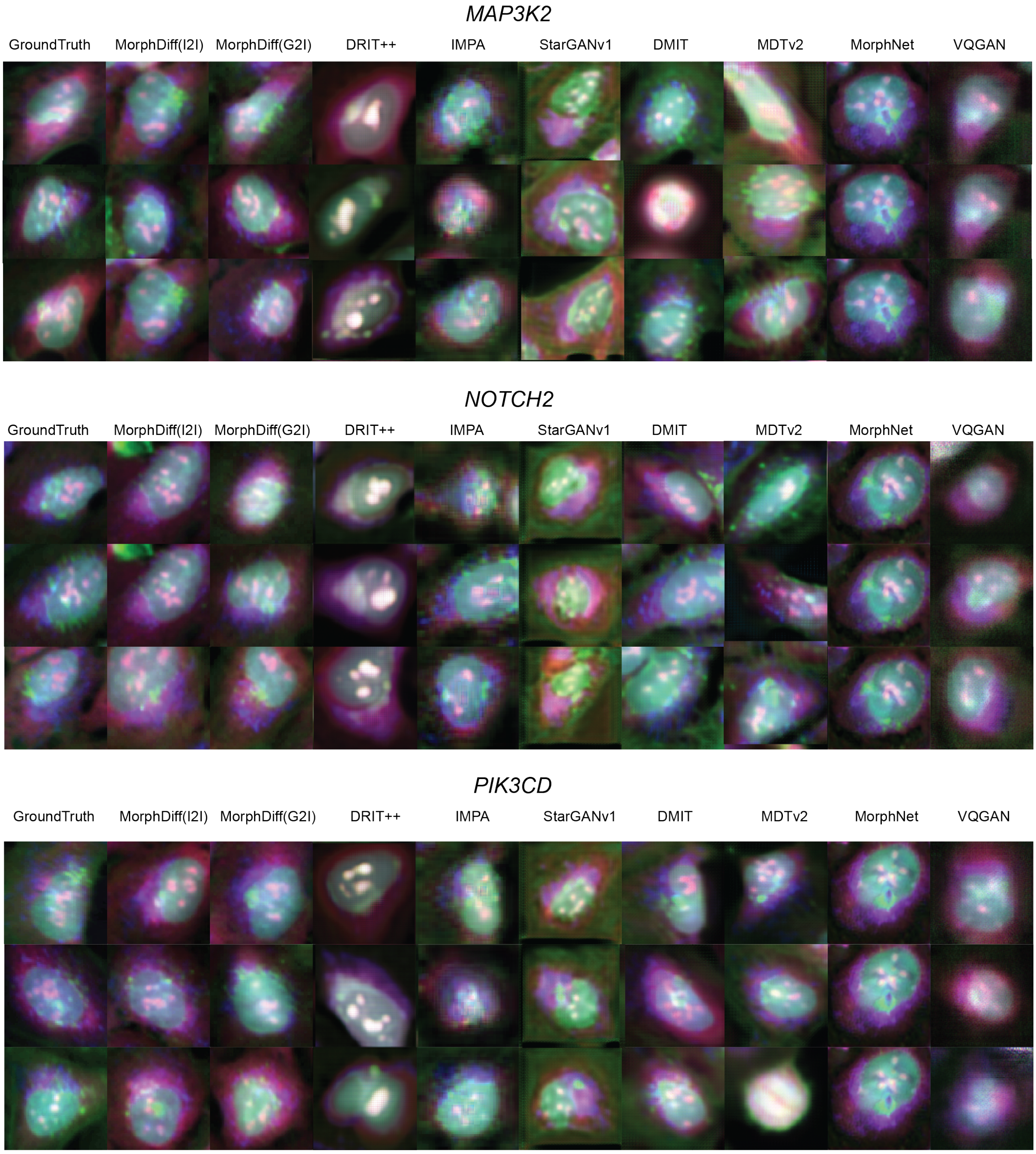

Supplementary Figure 2: Visualization of the generated images from each baseline method as well as the ground-truth from the JUMP dataset. We randomly chose three ground-truth images for each genetic perturbation, and select generated images of baseline by SSIM[10] for visualization. MorphDiff (both modes) stands out as the top performer in terms of visual quality and detail across all perturbations. DMIT, IMPA and MDTv2 offer decent performance but generally fall short in detail and clarity. MorphNet generates reasonable images with some detail, but lacks diversity, and the detail is inconsistent with the ground truth. DRIT++, StarGANv1 and VQGAN exhibit varying levels of quality, often resulting in less clear images. The visual quality can be inconsistent with ground truth, with some images appearing blurry. This figure visualizes the results for *MAP3K2*, *NOTCH2* and *PIK3C* genetic perturbations. The scale bar is 20 μm.

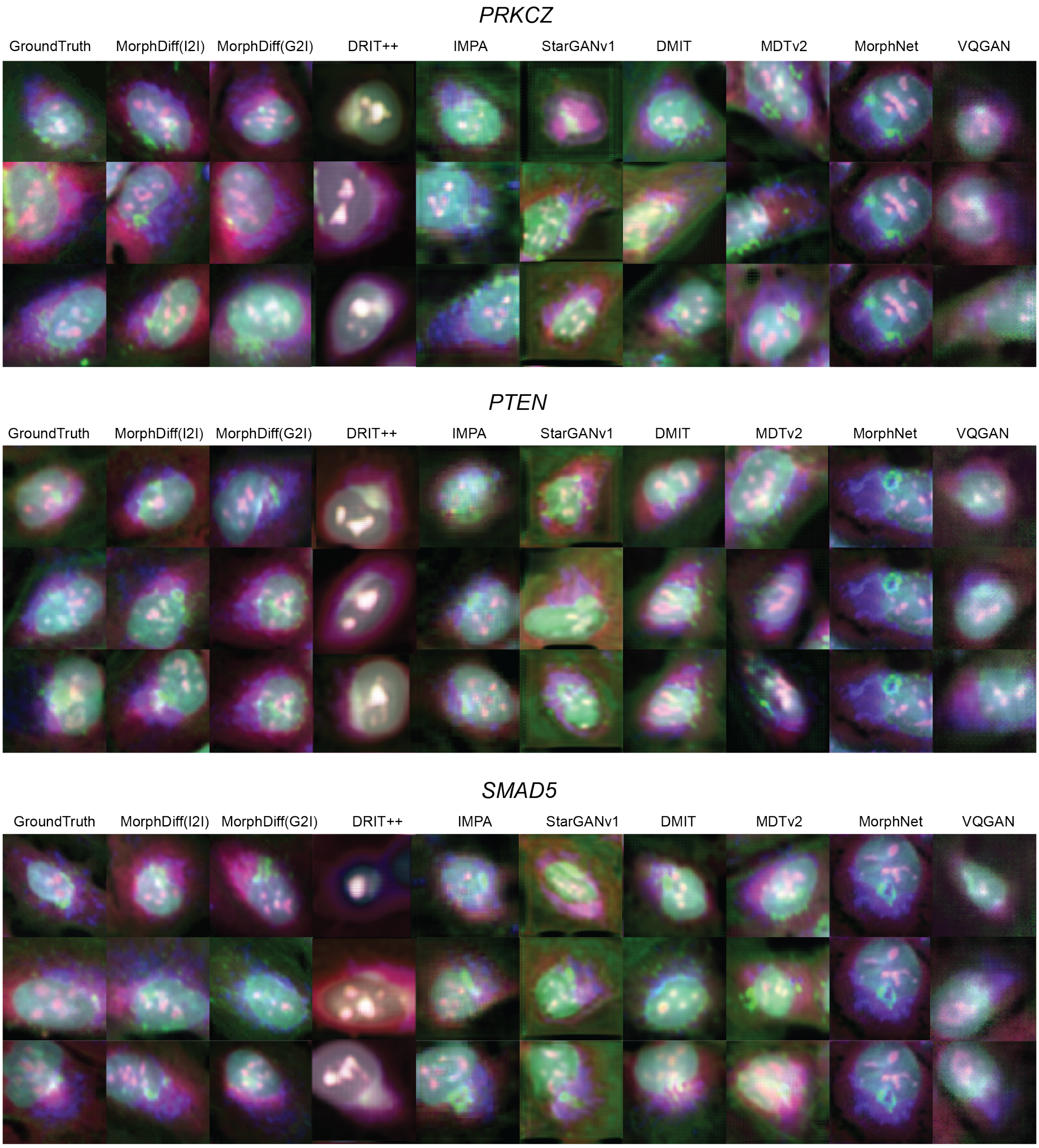

Supplementary Figure 3: Visualization of the generated images from each baseline method as well as the ground-truth from the JUMP dataset. We randomly chose three ground-truth images for each genetic perturbation, and select generated images of baseline by SSIM[10] for visualization. MorphDiff (both modes) stands out as the top performer in terms of visual quality and detail across all perturbations. DMIT, IMPA and MDTv2 offer decent performance but generally fall short in detail and clarity. MorphNet generates reasonable images with some detail, but lacks diversity, and the detail is inconsistent with the ground truth. DRIT++, StarGANv1 and VQGAN exhibit varying levels of quality, often resulting in less clear images. The visual quality can be inconsistent with ground truth, with some images appearing blurry. This figure visualizes the results for *PRKCZ*, *PTEN* and *SMAD5* genetic perturbations. The scale bar is 20 μm.

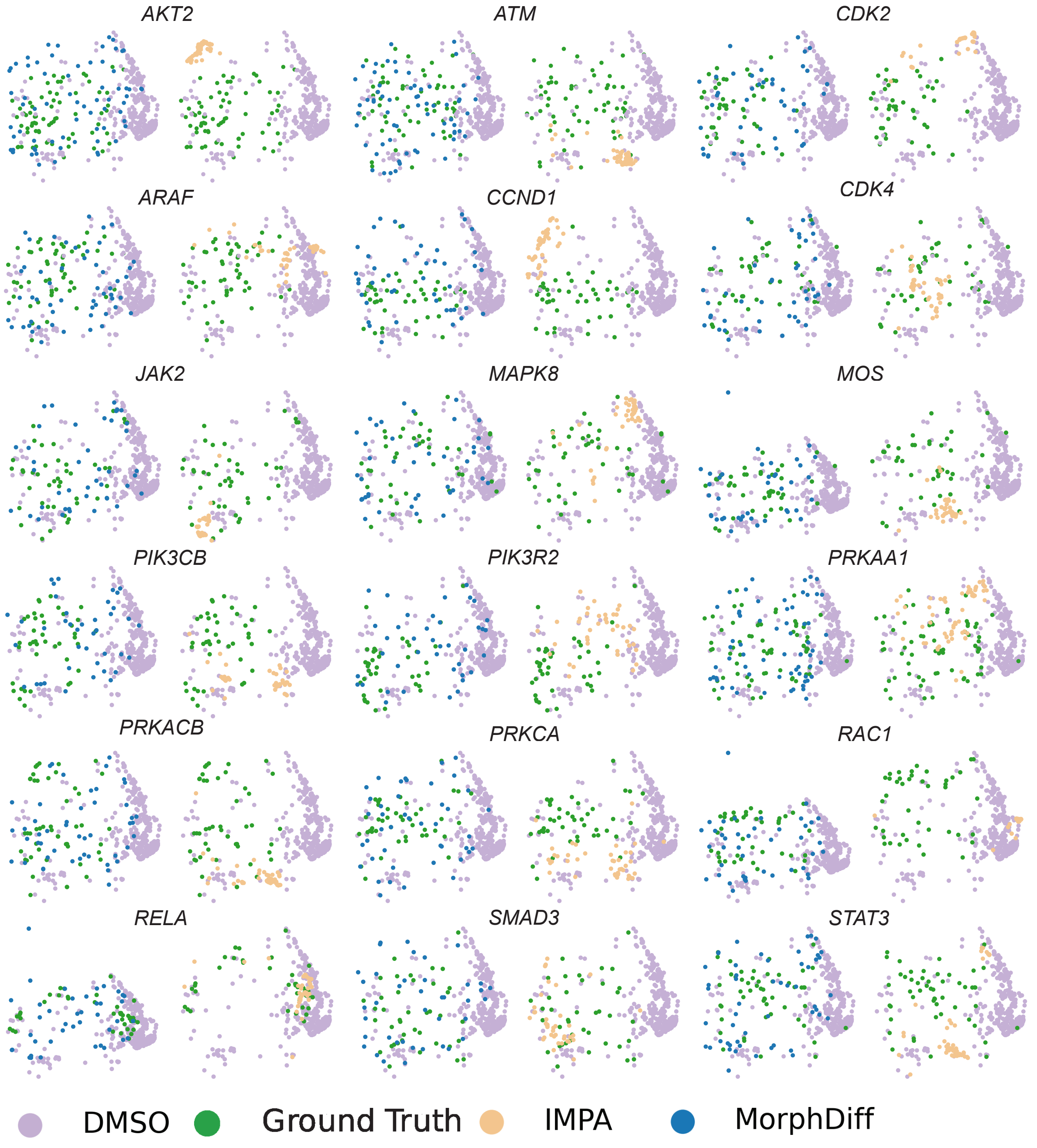

Supplementary Figure 4: UMAP visualization of the generated cell morphology CellProfiler feature on 18 genetic perturbations for IMPA and MorphDiff(G2I). These perturbation morphology images are selected from the perturbations with the most images. This Supplementary Figure corresponds to Figure 2c in the main text. Source data are provided as Source Data files.

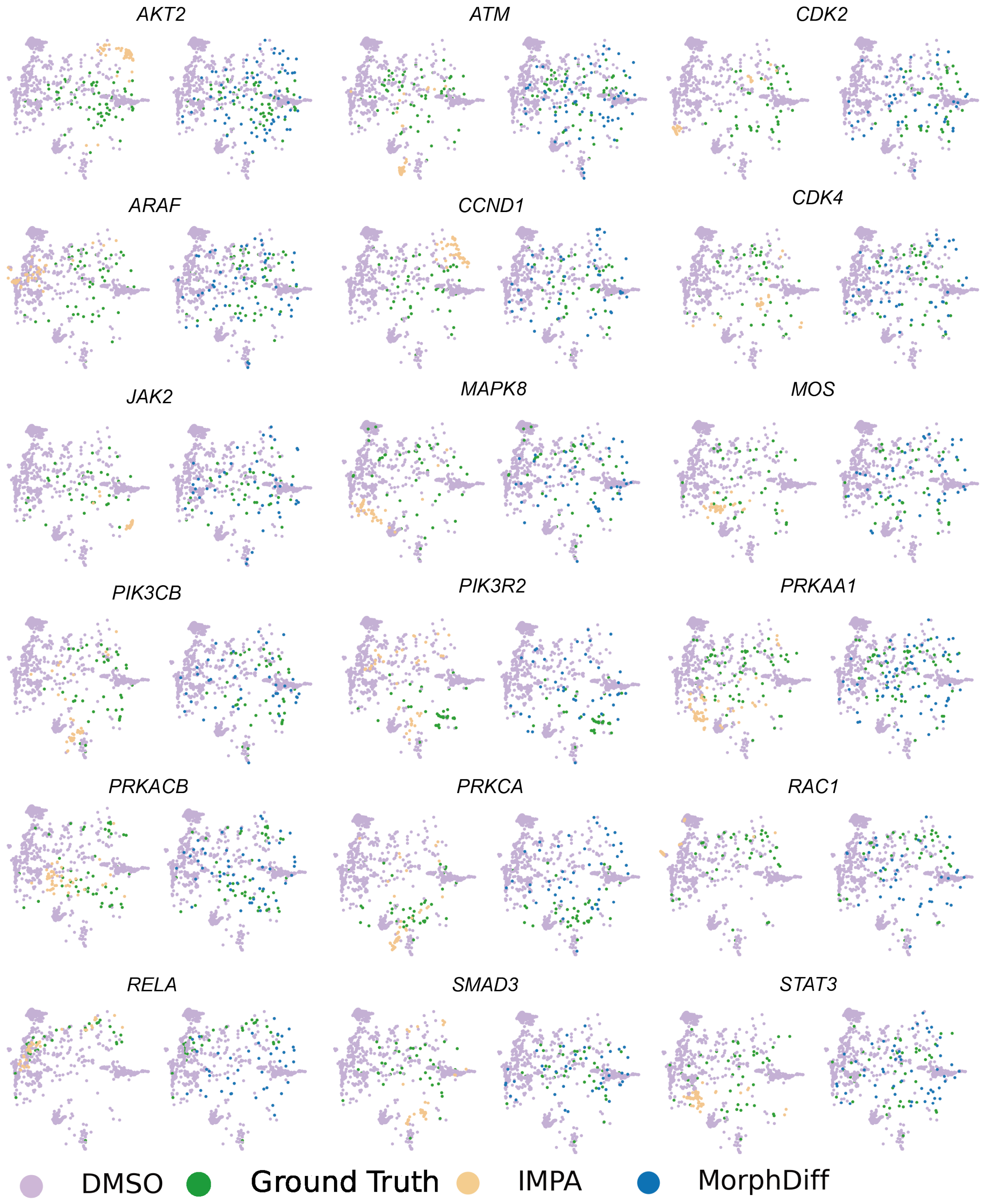

Supplementary Figure 5: UMAP visualization of the generated cell morphology CellProfiler feature on 18 genetic perturbations for IMPA and MorphDiff(I2I). These perturbation morphology images are selected from the perturbations with the most images. This Supplementary Figure corresponds to Figure 2c in the main text. Source data are provided as Source Data files.

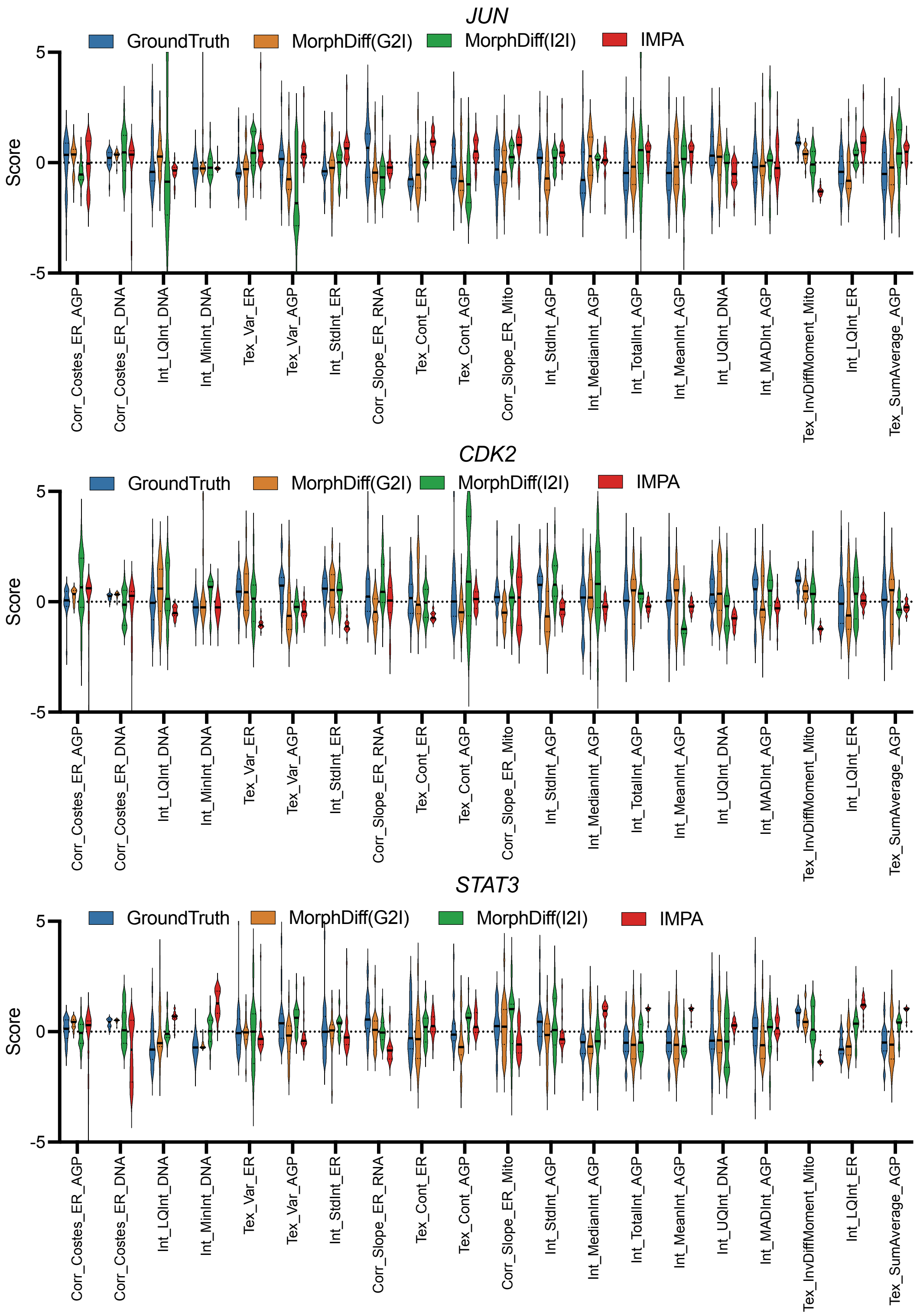

Supplementary Figure 6: Distribution of CellProfiler features between ground-truth, IMPA-generated, MorphDiff(G2I)-generated and MorphDiff(I2I)-generated morphology on *JUN*, *CDK2* and *STAT3* genetic perturbation. 'LQ' stands for 'LowerQuartile'. 'UQ' stands for 'UpperQuartile', 'InvDiffMoment' stands for 'InverseDifferenceMoment', 'Var' stands for 'Variance'. 'Tex' stands for 'Texture'. 'Corr' stands for 'correlation'. 'Int' stands for 'Intensity'. This Supplementary Figure corresponds to Figure 2d in the main text. Source data are provided as a Source Data file.

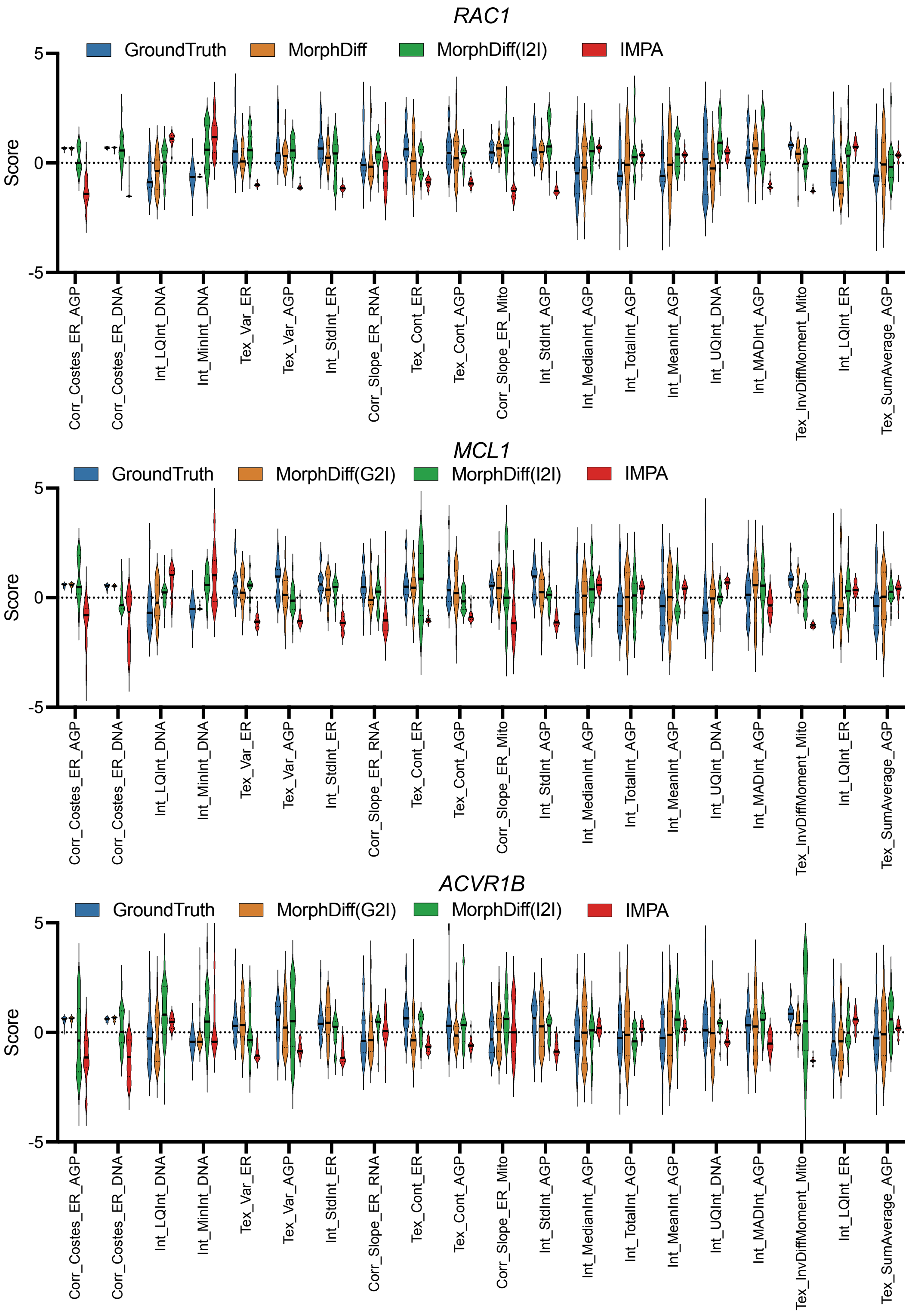

Supplementary Figure 7: Distribution of CellProfiler features between ground-truth, IMPA-generated, MorphDiff(G2I)-generated and MorphDiff(I2I)-generated morphology on *RAC1*, *MCL1* and *ACVR1B* genetic perturbation. 'LQ' stands for 'LowerQuartile'. 'UQ' stands for 'UpperQuartile'. 'InvDiffMoment' stands for 'InverseDifferenceMoment'. 'Var' stands for 'Variance'. 'Tex' stands for 'Texture'. 'Corr' stands for 'correlation'. 'Int' stands for 'Intensity'. This Supplementary Figure corresponds to Figure 2d in the main text. Source data are provided as a Source Data file.

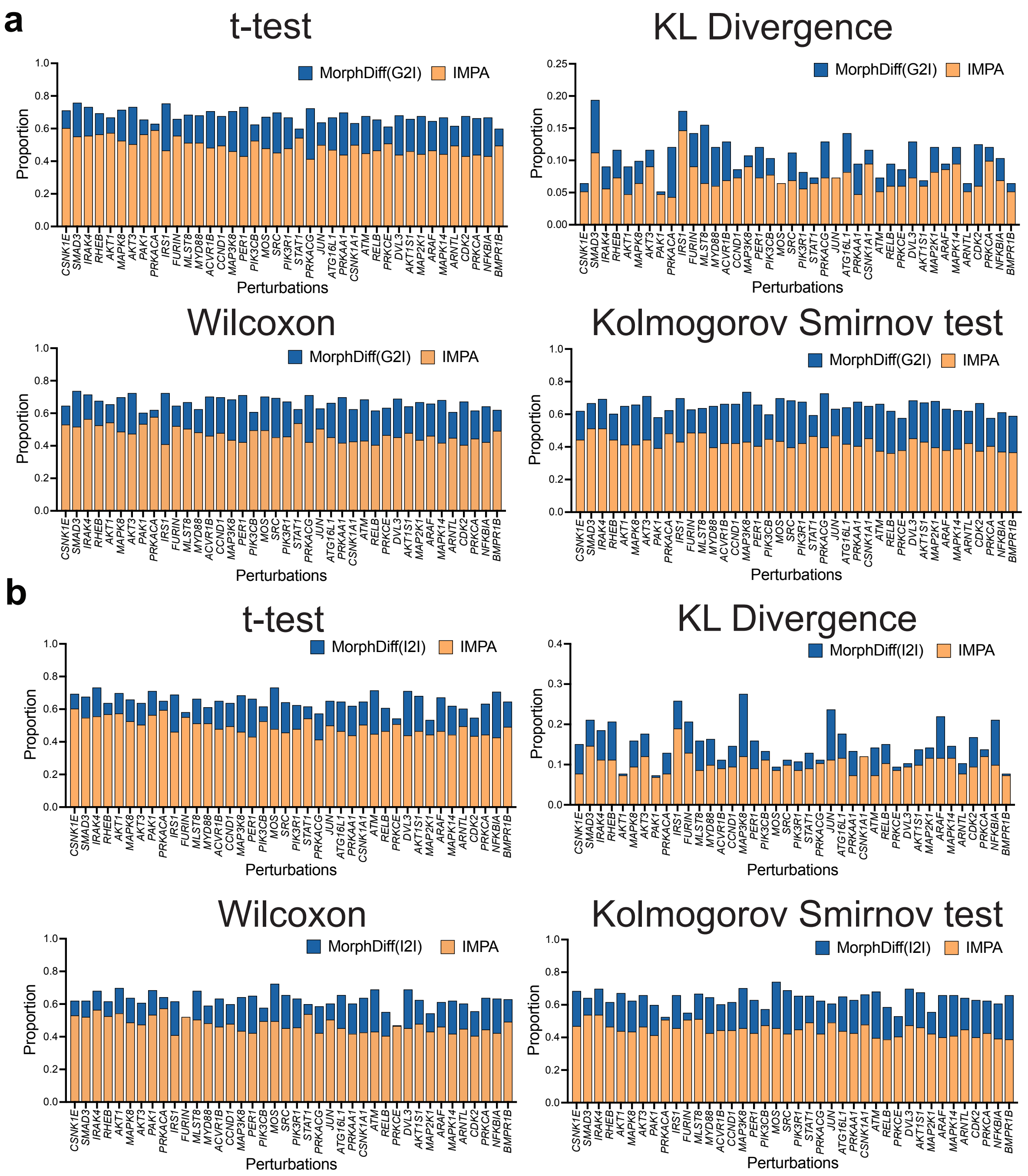

Supplementary Figure 8: **a.** Using additional tests for Figure 2e in the manuscript. t-test (two-sided), KL-divergence metric (with a threshold of 3, i.e., KL divergence lower than 3 means the distribution is similar), Wilcoxon signed-rank test (two-sided and same as in maintext), Kolmogorov Smirnov test (two-sided). Source data are provided as a Source Data file. **b.** Similar to **a**, but the results are compared between MorphDiff(I2I) and IMPA. Source data are provided as a Source Data file.

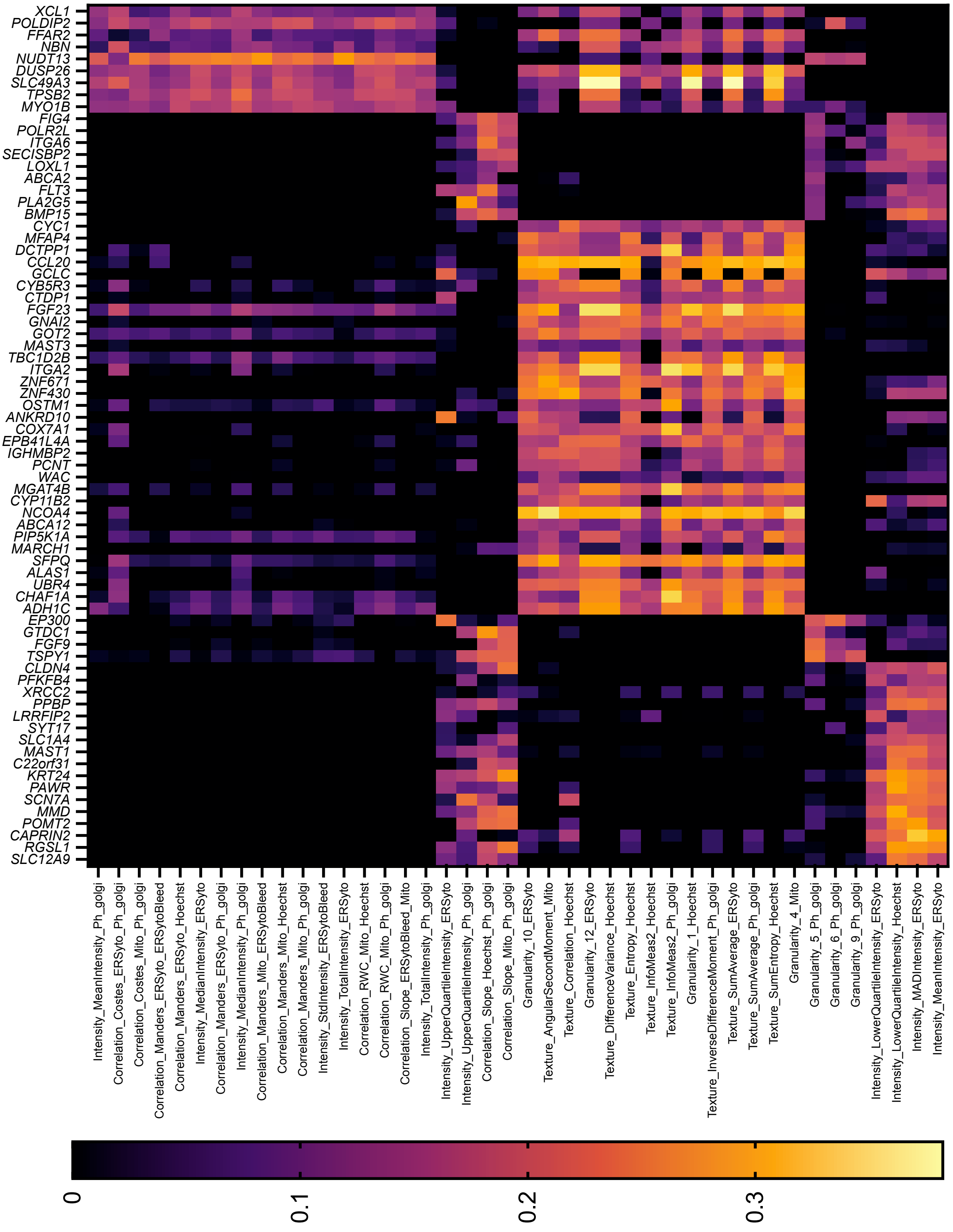

Supplementary Figure 9: Correlation heatmap between the gene expression and the CellProfiler Features from the ground-truth cell morphology.

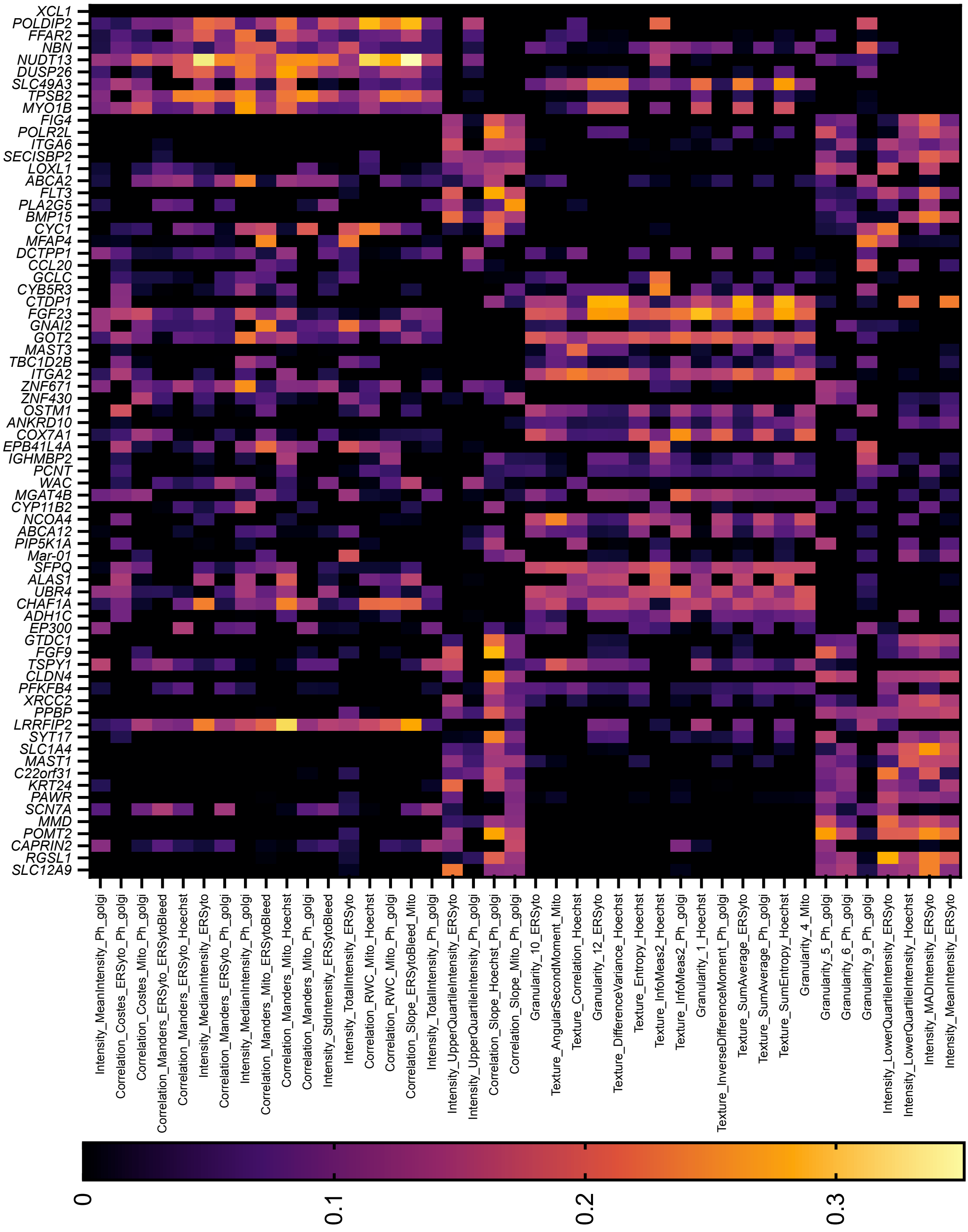

Supplementary Figure 10: Correlation heatmap between the gene expression and the CellProfiler Features from the predicted cell morphology of MorphDiff(G2I).

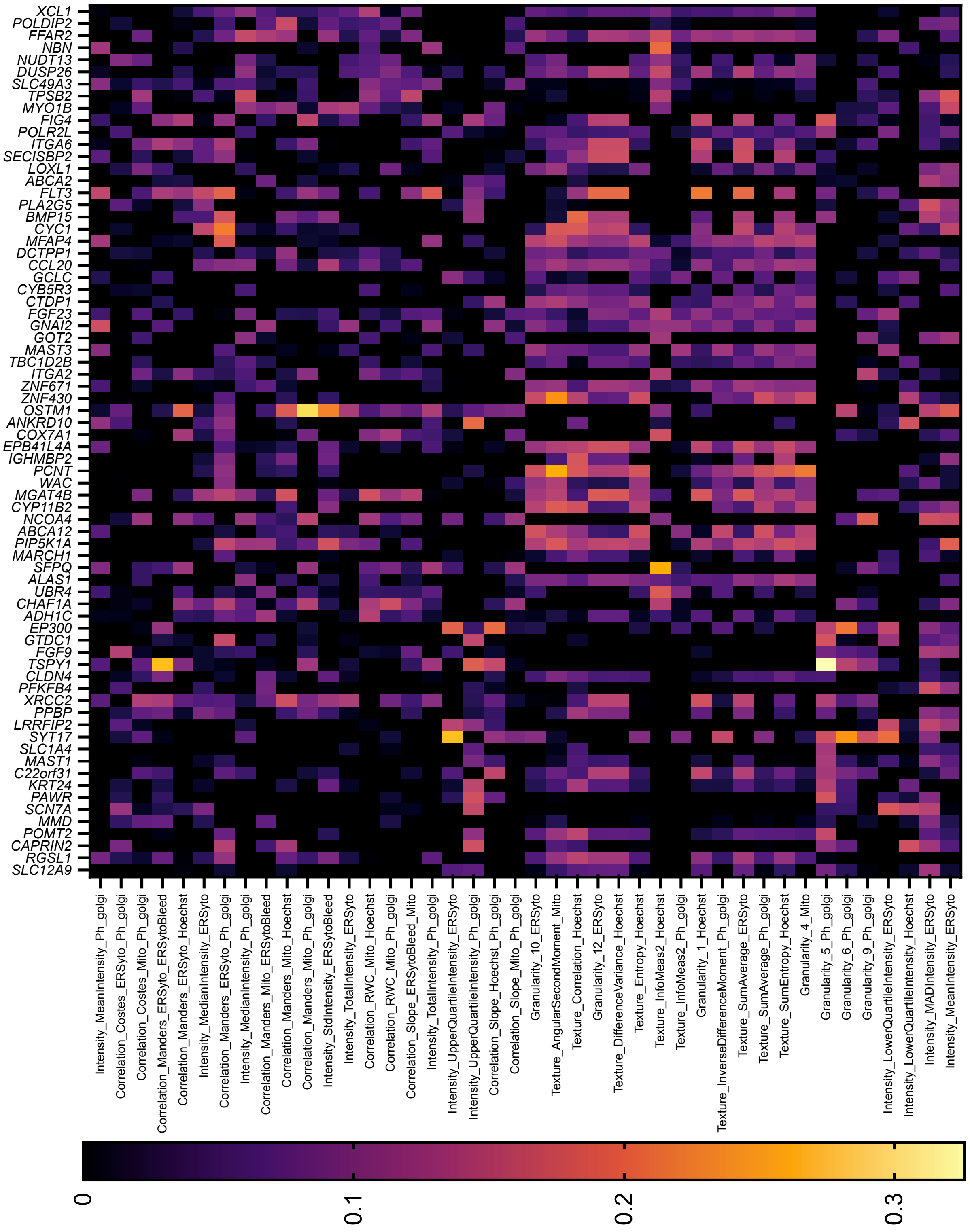

Supplementary Figure 11: Correlation heatmap between the gene expression and the CellProfiler Features from the predicted cell morphology of IMPA.

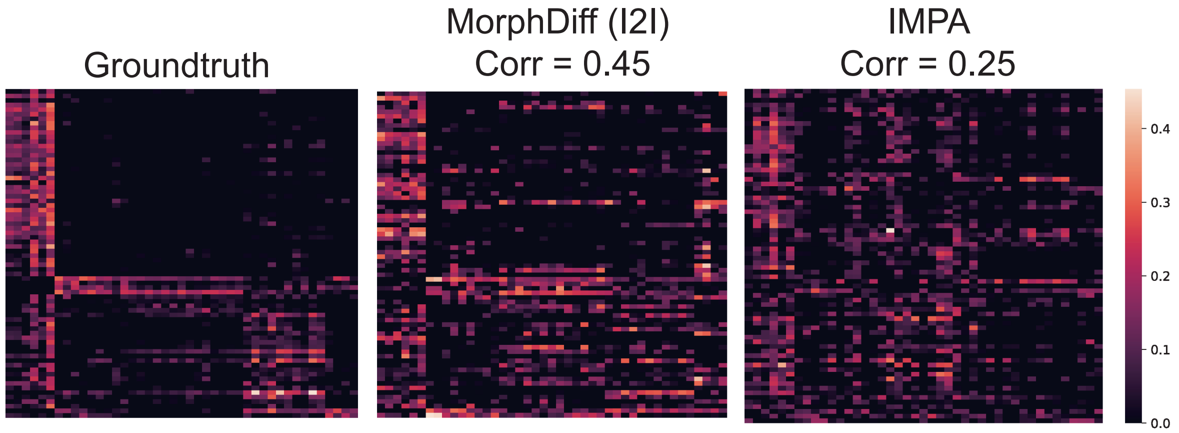

Supplementary Figure 12: The heatmap of the correlation between the CellProfiler features of ground-truth morphology, MorphDiff(I2I)-generated morphology, and IMPA-generated morphology with L1000 gene expression.

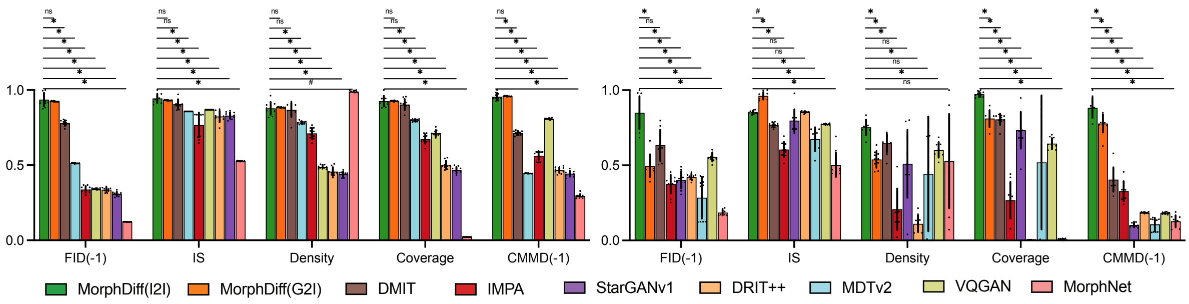

Supplementary Figure 13: Leave-one-out validation performance on 10 MOAs and 10 targets on the A549 cell line. The significance test used here is the Wilcoxon signed-rank test, corrected with the Bonferroni correction. Star ('*') means that MorphDiff(I2I) surpasses the methods with a p-value less than 0.05. The hashtag ('#') represents the situation in which the method outperforms MorphDiff(I2I) significantly. 'ns' means no significant difference. **a.** Leave-one-out validation on the LINCS Target leave-one-out set. **b.** Leave-one-out validation on the LINCS MOA leave-one-out set. The results show that MorphDiff(both modes) rank the best, including cases in which they share the first place with other methods. Source data are provided as a Source Data file.

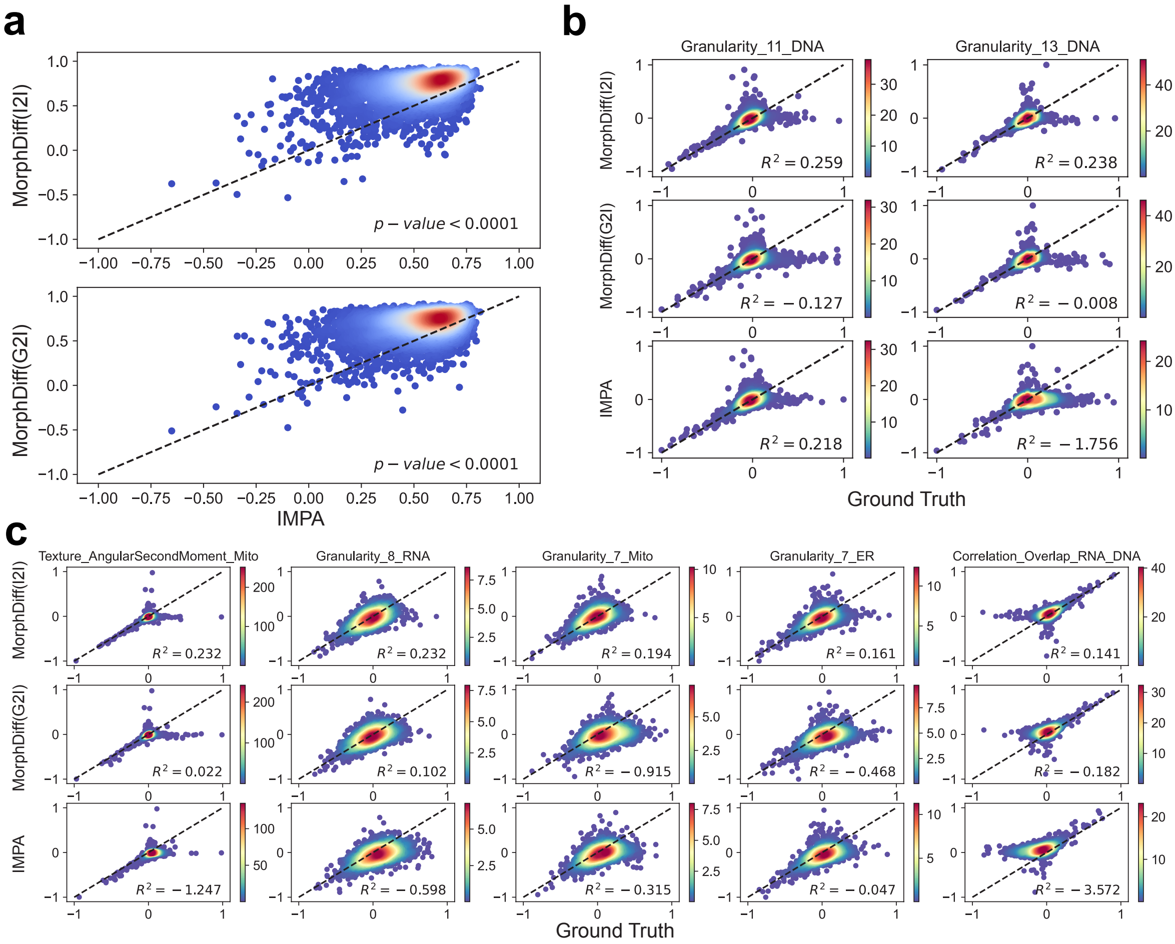

Supplementary Figure 14: **a.** The R^2 score between the ground-truth CellProfiler feature vectors and the generated CellProfiler feature vectors on the CDRP OOD set. The x-axis represents the R^2 scores between IMPA and ground truth for each sample. The y-axis represents the R^2 scores of MorphDiff(G2I) and MorphDiff(I2I) against ground truth respectively. The p-values calculated by one-sided Wilcoxon signed-rank test indicate the significance of the distribution of the y-axis being greater than that of the x-axis. 89.6% of the generated points from MorphDiff(I2I) have an R^2 value greater than 0.5, 23.0% greater than 0.8, compared to 89.0% and 15.7 % for MorphDiff(G2I), and 65.8% and 0% for IMPA, respectively. **b.** The difference between control and perturbations for selected CellProfiler features on the CDRP ID set. **c.** The difference between control and perturbations for another 5 selected CellProfiler features on the CDRP ID set. Source data are provided as a Source Data file.

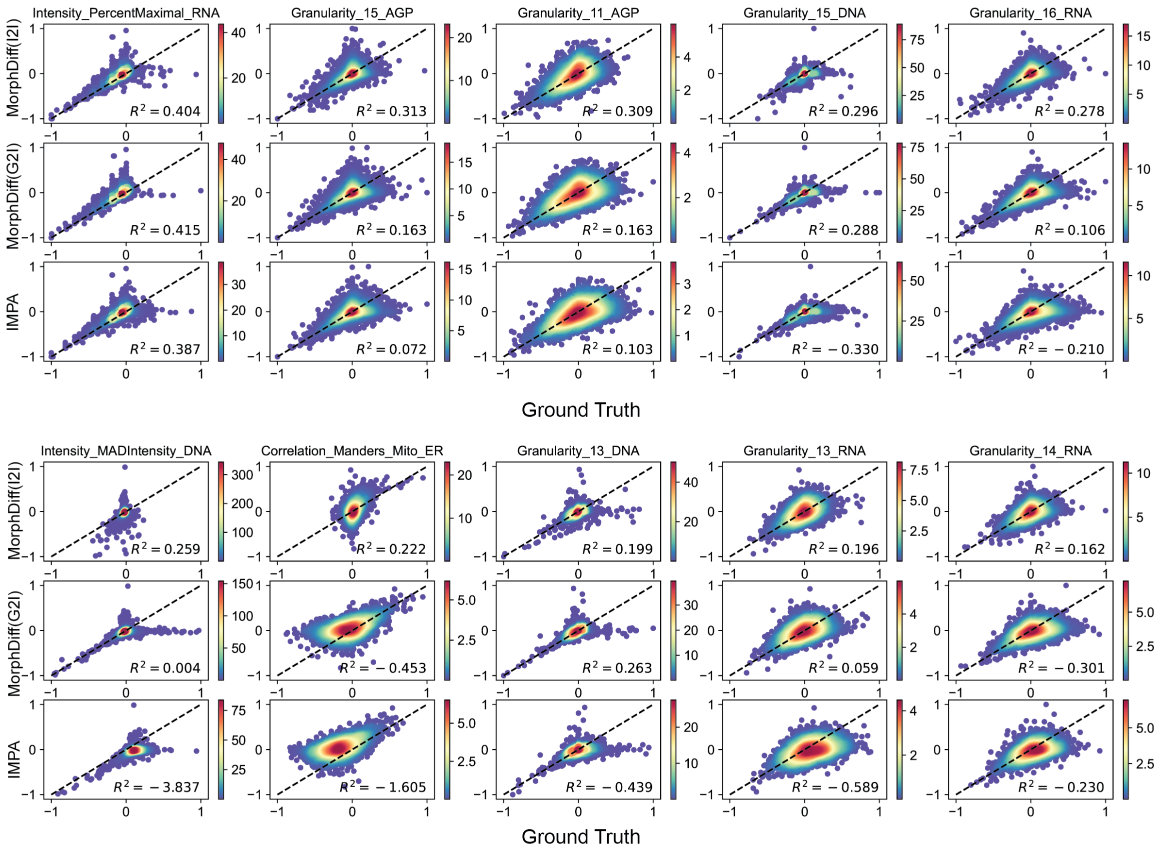

Supplementary Figure 15: Predictions on morphological changes between control and perturbations for selected CellProfiler features on the CDRP OOD set. The x-axis displays the changes between the ground-truth perturbations and the control, while the y-axis displays the changes in the generated perturbations and the control. The R^2 signifies the degree of fit between ground-truth and generated changes. Source data are provided as a Source Data file.

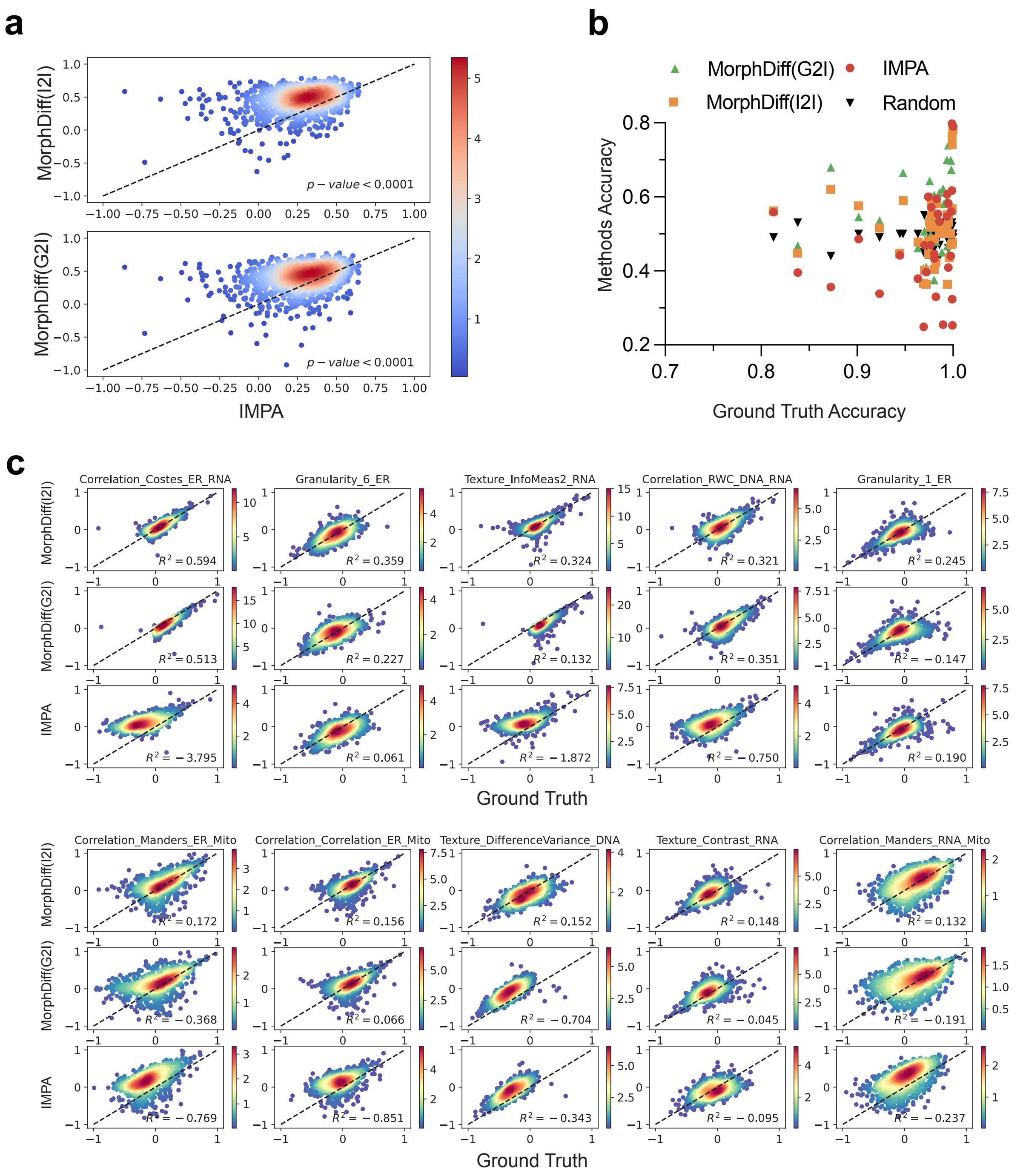

Supplementary Figure 16: The analysis of CellProfiler features on LINCS leave-one-out dataset. **a.** The x-axis represents the R^2 scores between IMPA and ground truth. The y-axis represents the R^2 scores of MorphDiff. The $p-$ values of one-sided Wilcoxon signed-rank test indicate the significance of the distribution of the y-axis being greater than that of the x-axis. 45% of the generated points from MorphDiff(I2I) have an R^2 value greater than 0.5, while 38.8% of MorphDiff(G2I) and 9.2% of IMPA exceed 0.5. **b.** The x-axis represents the ground-truth classification accuracy between different pairs of perturbations, and the y-axis means the classification accuracy on generated perturbations. The average accuracy scores for the ground truth, MorphDiff(I2I), MorphDiff(G2I), and IMPA are 97.3%, 55.9%, 59.9%, and 49.3%, respectively. The one-sided Wilcoxon signed-rank test results are as follows: MorphDiff(I2I) vs. IMPA: $p < 0.05$; MorphDiff(G2I) vs. IMPA: $p < 0.001$; MorphDiff(G2I) vs. MorphDiff(I2I): $p < 0.001$; MorphDiff(I2I) vs. Random: $p < 0.05$; MorphDiff(G2I) vs. Random: $p < 0.001$; IMPA vs. Random: $p = 0.59$. **c.** The x-axis displays the changes between the ground-truth perturbations and the control, while the y-axis displays the changes in the generated perturbations and the control. The R^2 signifies the degree of fit between ground-truth and generated changes. Source data are provided as a Source Data file.

a

Compounds	SMILE
BRD-K02078126	COc1ccc(CNC(=O)C[C@@H]2CC[C@@H](NC(=O)CN(C)C)[C@H](CO)O2)cc1
BRD-K29850806	Cc1ccccc1-n1c(=O)cc(NCc2ccc3OCOc3c2)[nH]c1=O
BRD-K05060234	COc1ccc(CNC(=O)C[C@H]2CC[C@@H](NC(=O)CN(C)C)[C@H](CO)O2)cc1
BRD-K56572622	Cc1cc(C)c(C#N)c(Nc2ccc3OCCOc3c2)n1
BRD-K16255400	CCCNC(=O)N[C@H]1C=C[C@H](CC(=O)NC2CCCCC2)O[C@@H]1CO
BRD-K81322049	[O-][N+](=O)c1ccccc1CSc1nc2ccccc2o1
BRD-K74817844	CN(C)CCCNC(=O)C[C@H]1CC[C@@H](NC(=O)c2ccccc2F)[C@H](CO)O1
BRD-K74486924	OC[C@@H]1O[C@H](CC(=O)Nc2ccccc2)C=C[C@@H]1NC(=O)Nc1cc(F)ccc1F
BRD-K78074905	COc1ccccc1CNC(=O)c1ccc2c(OCC=C)n(C)nc2c1
BRD-K37912617	OC[C@@H]1O[C@H](CCNC(=O)CN2CCOCC2)CC[C@@H]1NC(=O)Cc1ccccn1

b

Compounds	SMILE
acrivastine	Cc1ccc(/C(=C\CN2CCCC2)c2cccc(/C=C/C(=O)O)n2)cc1
arry-334543	CC1COC(=N1)NC2=CC3=C(C=C2)N=CN=C3NC4=CC(=C(C=C4)OCC5=NC=CS5)Cl
balicatib	CCCN1CCN(CC1)C2=CC=C(C=C2)C(=O)NC3(CCCCC3)C(=O)NCC#N
cp-724714	CC1=NC=C(C=C1)OC2=C(C=C(C=C2)NC3=NC=NC4=C3C=C(C=C4)C=CCNC(=O)COC)C
homochlorcyclizine	CN1CCCN(C(c2ccccc2)c2ccc(Cl)cc2)CC1
ketotifen	CN1CCC(=C2C3=C(C(=O)CC4=CC=CC=C42)SC=C3)CC1
levomequitazine	c1ccc2c(c1)Sc1ccccc1N2C[C@H]1CN2CCC1CC2
odanacatib	CC(C)(CC(C(=O)NC1(CC1)C#N)NC(C2=CC=C(C=C2)C3=CC=C(C=C3)S(=O)(=O)C)C(F)(F)F)F
otamixaban	COC(=O)[C@H](Cc1cccc(C(=N)N)c1)[C@@H](C)NC(=O)c1ccc(-c2cc[n+]([O-])cc2)cc1
rivaroxaban	C1COCC(=O)N1C2=CC=C(C=C2)N3CC(OC3=O)CNC(=O)C4=CC=C(S4)Cl

Supplementary Figure 17: **a.** The drugs analyzed in manuscript Figure 3d. **b.** The drugs analyzed in Supplementary Figure 16b.

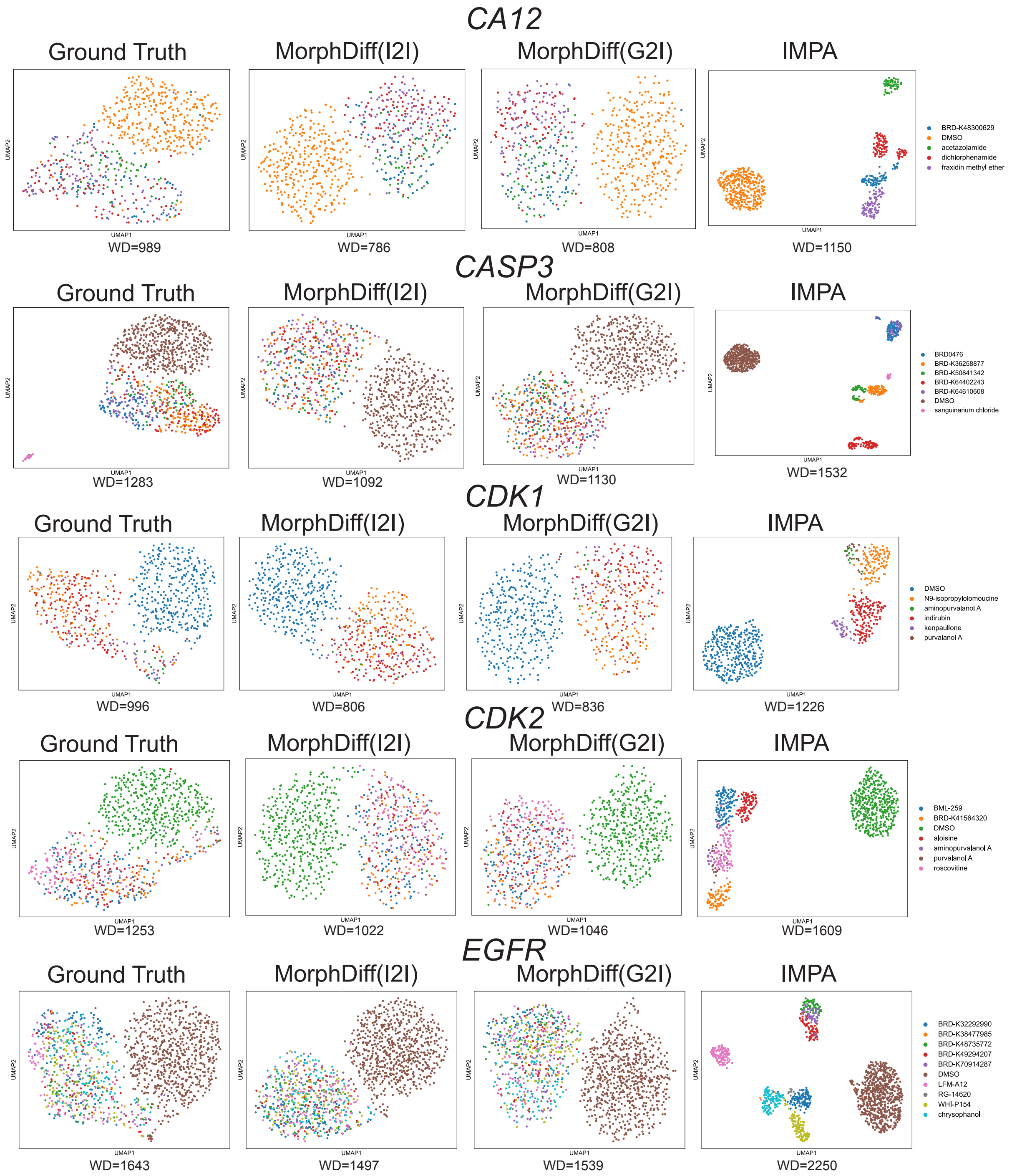

Supplementary Figure 18: MorphDiff predicts morphological changes on the target level. Morphological features were extracted from both ground-truth and generated images. The perturbed morphological features can be distinguished from the control, and the drugs targeting the same target cause similar changes in morphological space. The images generated by MorphDiff(I2I) and MorphDIff(G2I) are consistent with this distinction, while IMPA generates different clusters consisting of different perturbations. WD represents the Wasserstein Distance between perturbed and control CellProfiler features. The WD of MorphDiff is also closer to ground truth. Source data are provided as a Source Data file.

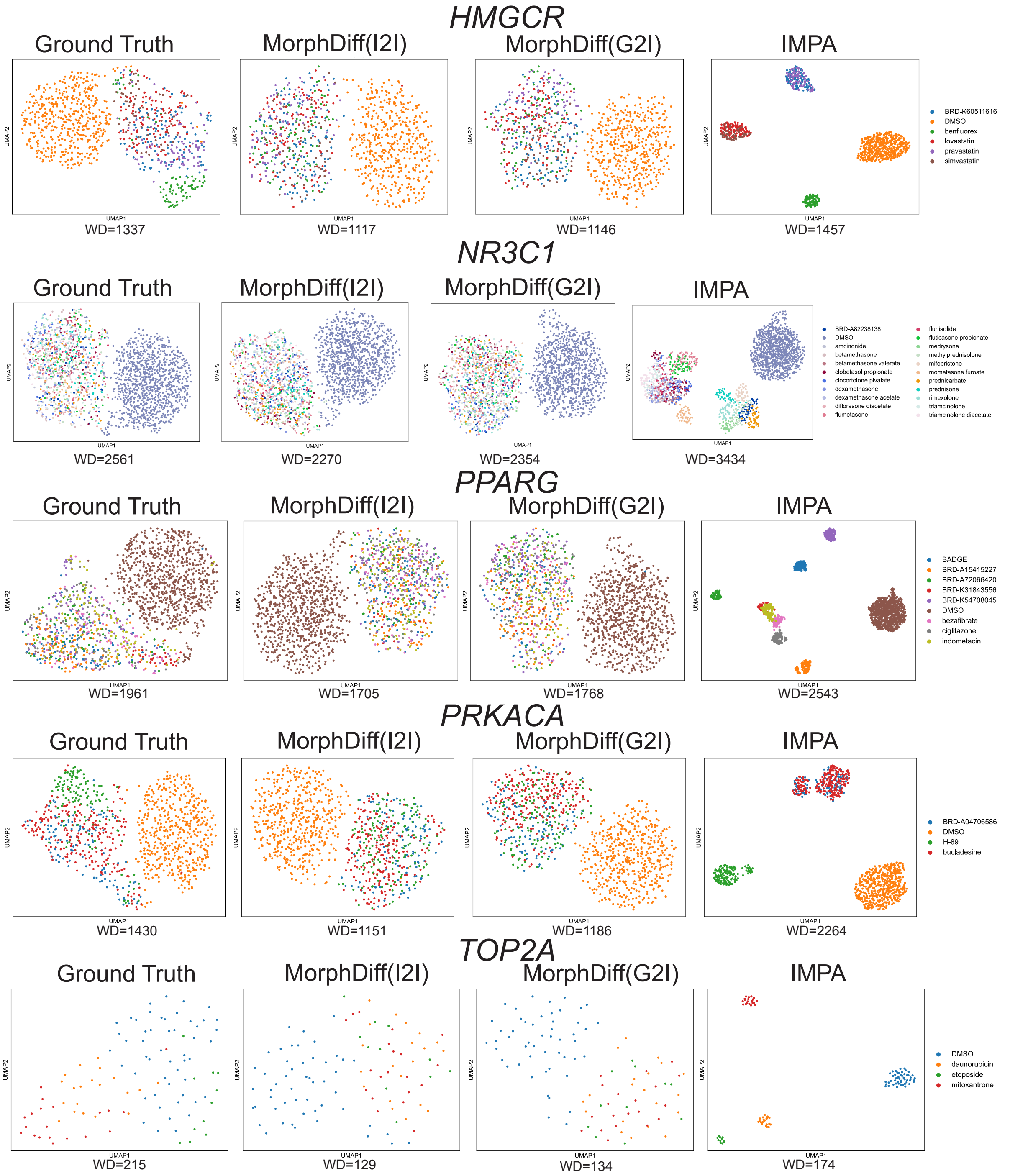

Supplementary Figure 19: MorphDiff predicts morphological changes on the target level. CellProfiler features were extracted from both ground-truth and generated images for the other five targets. WD represents the Wasserstein Distance between perturbed and control CellProfiler features. The WD of MorphDiff is also closer to ground truth. Source data are provided as a Source Data file.

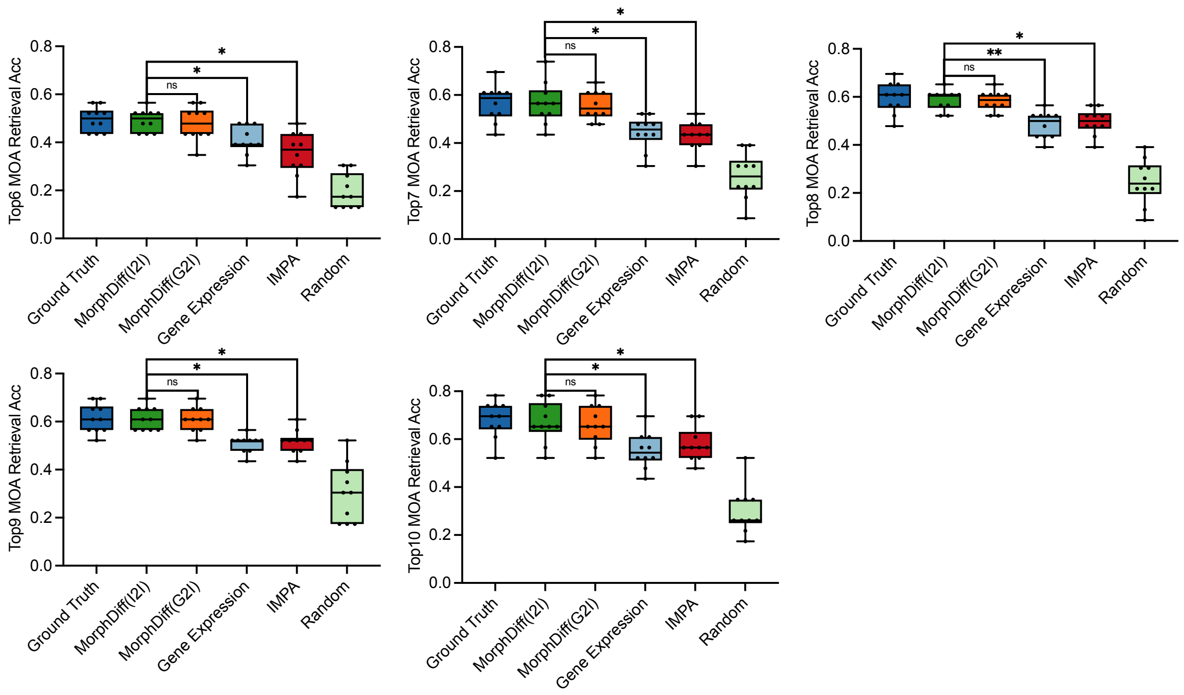

Supplementary Figure 20: Ablation study on the number of k in the top k MOA retrieval as described in Figure 4e in the manuscript. Source data are provided as a Source Data file.

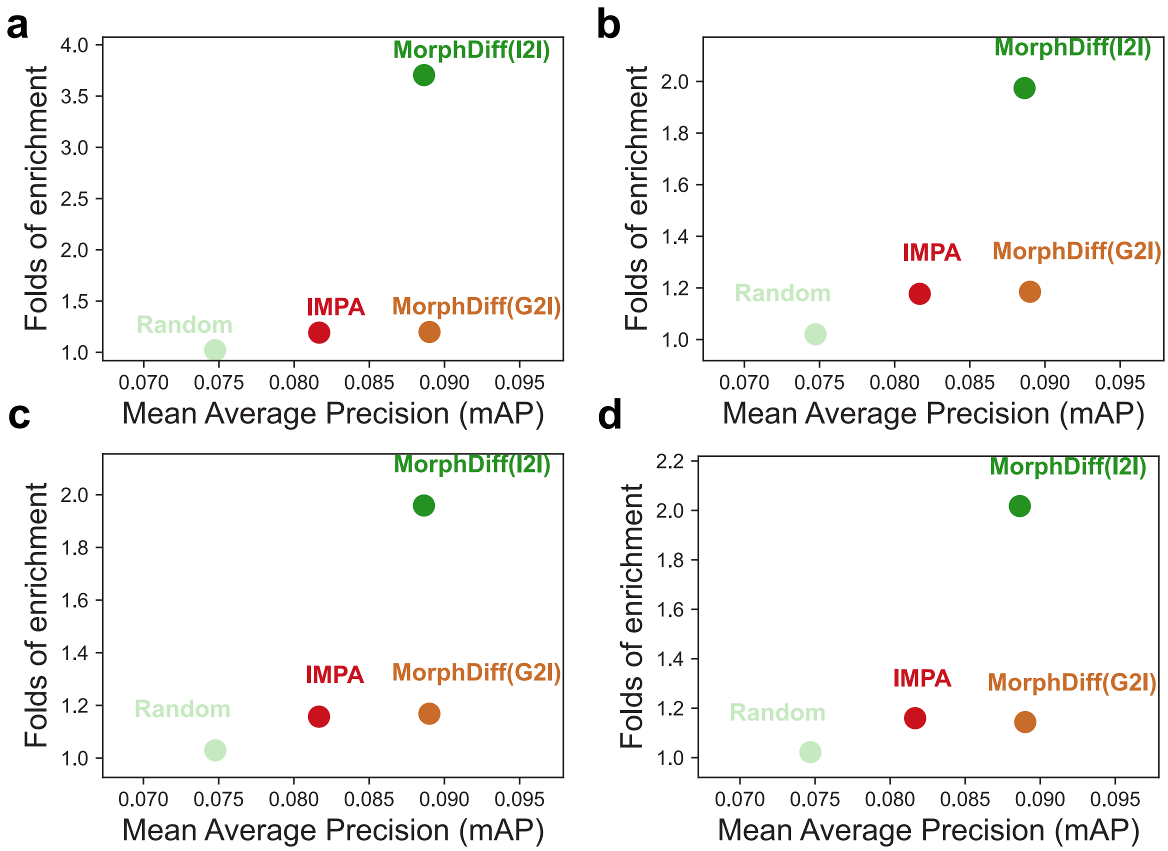

Supplementary Figure 21: Performance of generated feature representations for the methods according to two metrics: Mean Average Precision (mAP) in the x-axis and Folds of Enrichment in the y-axis (see Methods). Each point indicates the mean of these metrics over all queries using the DeepProfiler embeddings. Each figure represents the performance of different thresholds for folds of enrichment analysis. **a.** The threshold is 0.95 (top 5%). **b.** The threshold is 0.97 (top 3%). **c.** The threshold is 0.98 (top 2%). **d.** The threshold is 0.99 (top 1%). Source data are provided as a Source Data file.

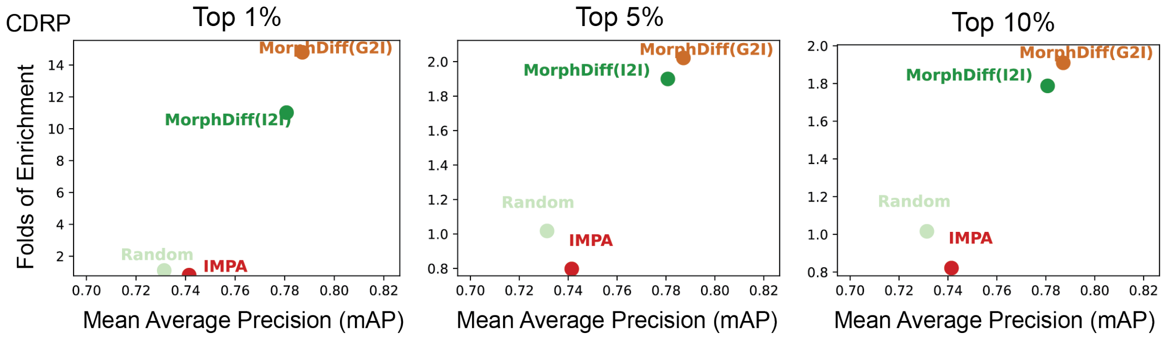

Supplementary Figure 22: Exploration analysis on generating cell morphology images with the L1000 gene expression profile on those perturbations without corresponding ground-truth cell morphology images. Folds of enrichment and mean average precision are used as evaluation metrics and the drug MOA is used as labels. The evaluation is conducted on the CDRP dataset. Top 1%, 5%, 10% refer to different threshold. Source data are provided as a Source Data file.

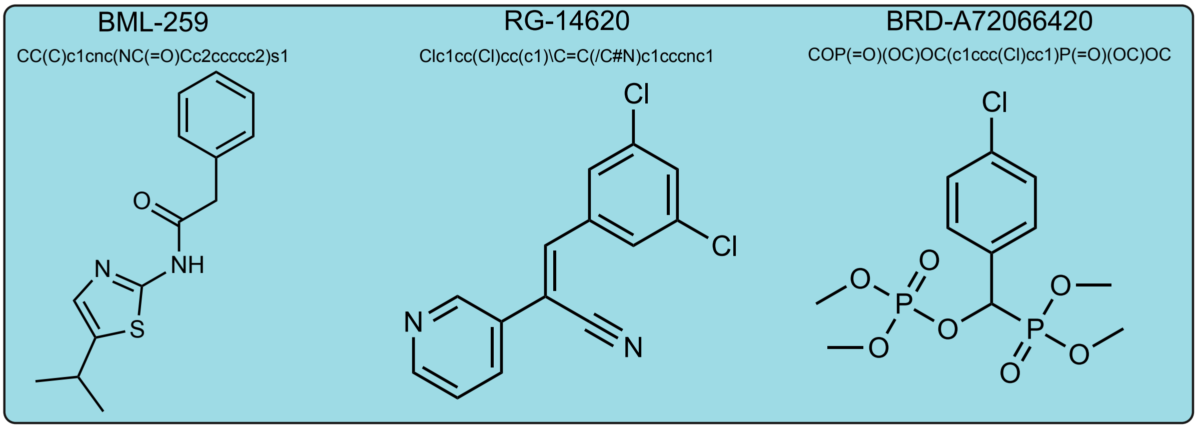

Supplementary Figure 23: The drug structures of BML-259, RG-14620, and BRD-A72066420.

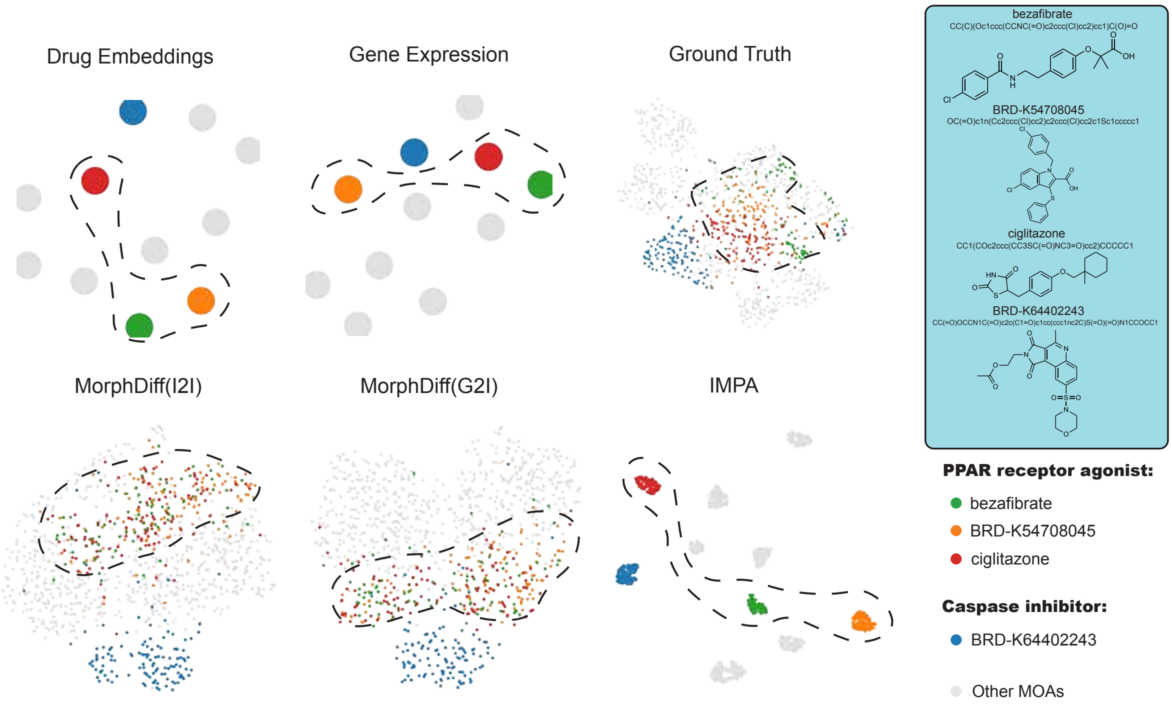

Supplementary Figure 24: The UMAP projection for different data, including drug embeddings, gene expression and DeepProfiler embeddings. The DeepProfiler embeddings are extracted from ground-truth images, generated images obtained from three methods respectively. The structures of bezafibrate, BRD-K54708045, ciglitazone and BRD-K64402243 are generated by ChemDraw. Source data are provided as a Source Data file.

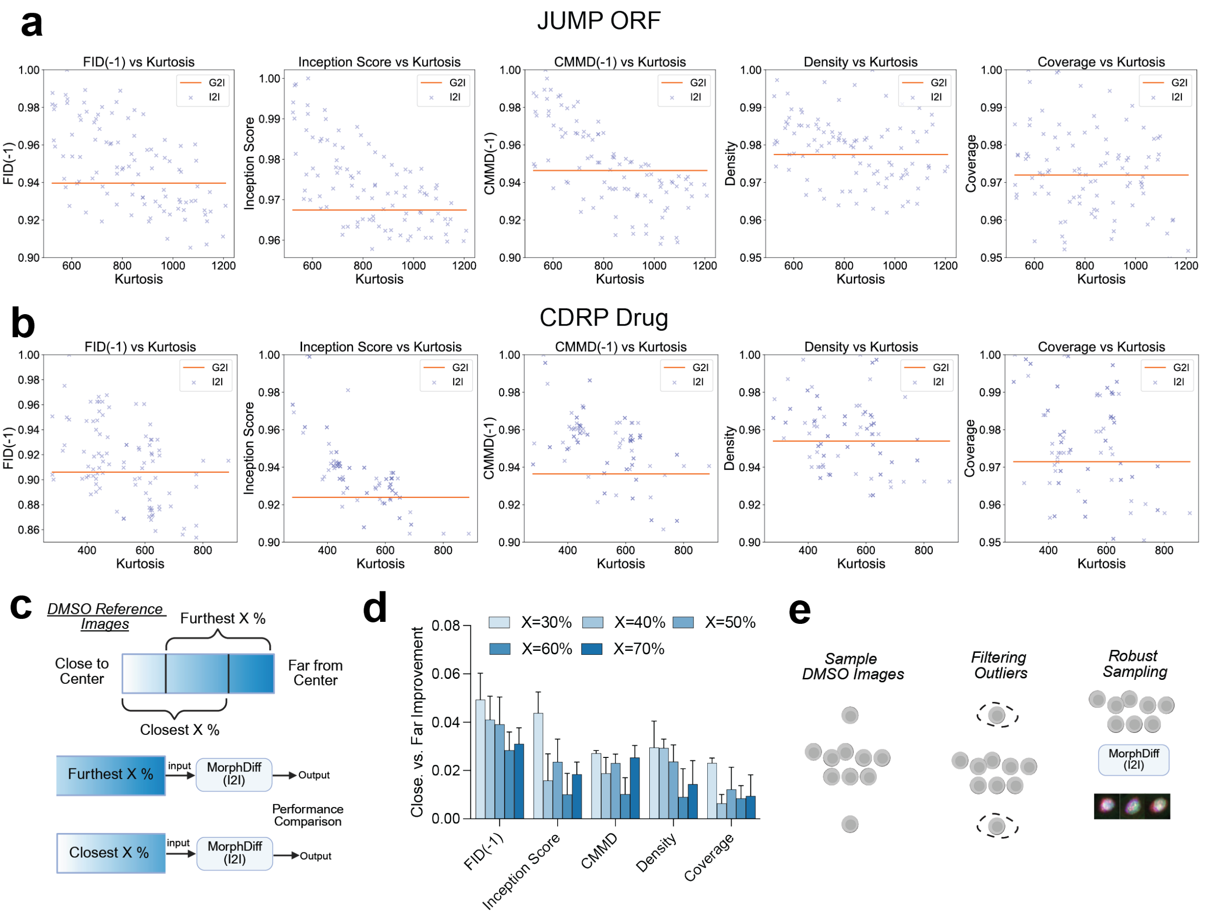

Supplementary Figure 25: Analysis of the potential impact of the quality of the DMSO control morphology images on the performance of the I2I mode of MorphDiff as well as practical tips. **a. b.** Analysis of the potential impact of the kurtosis of the DMSO control morphology images on the performance of the I2I mode of MorphDiff. For each point (blue cross), the x-axis represents the kurtosis of a reference set of DMSO control morphology images, and the y-axis indicates the corresponding performance metrics of the inference output using this set of reference images (I2I mode). The orange line indicates the performance of the G2I mode of MorphDiff, and the green dot/line indicates the kurtosis and performance of the DMSO reference images in the previous manuscript. We conducted the same analysis for the JUMP ORF dataset (a) and CDRP drug dataset (b) **c.** Illustration of our approach to improving the robustness of MorphDiff's I2I mode. We quantified the Mahalanobis distance between each DMSO sample and the center of the DMSO reference image set. We then compared the generative performance of MorphDiff(I2I) when using two different subsets: (1) the closest to center $X\%$ samples (least likely to be outliers) versus (2) the furthest to center $X\%$ samples (most likely to be outliers). Created in BioRender. Group, A. (2025) https://BioRender.com/w46s30j. **d.** Performance Illustration of the proposed filtering approach. y-axis means performance improvement by calculating the performance difference between the sampling output of the Closest $X\%$ and the Furthest $X\%$. **e.** Practical guidelines in applying the MorphDiff(I2I) in practice. It is suggested to first filter the outliers in the sample DMSO images therefore the sampling could be more robust. Created in BioRender. Group, A. (2025) https://BioRender.com/ro7qmth. Source data are provided as a Source Data file.

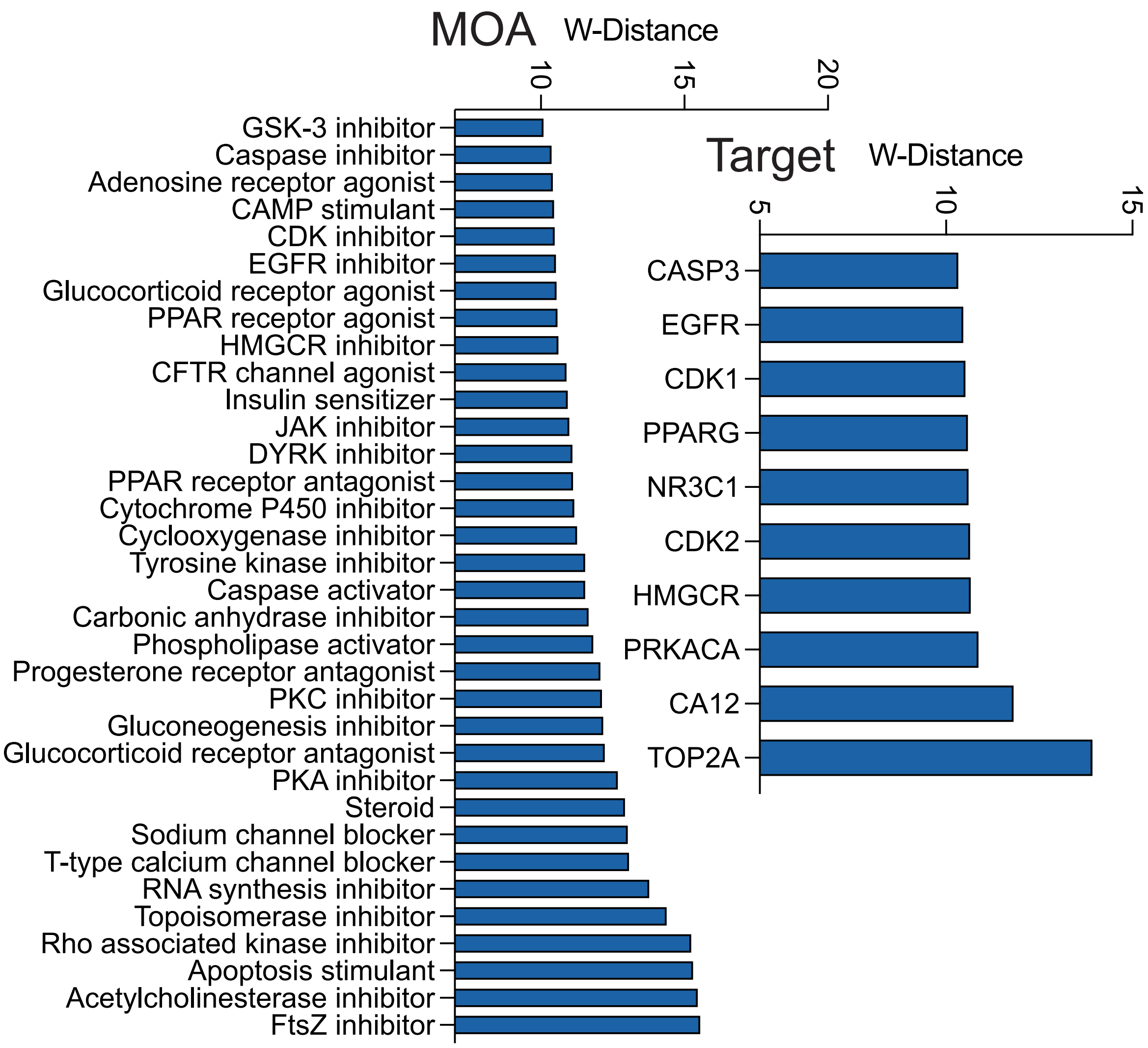

Supplementary Figure 26: Statistics showing the Wasserstein Distance between the DeepProfiler embeddings of the training dataset and the MOA/target set in the CDRP dataset. Source data are provided as a Source Data file.

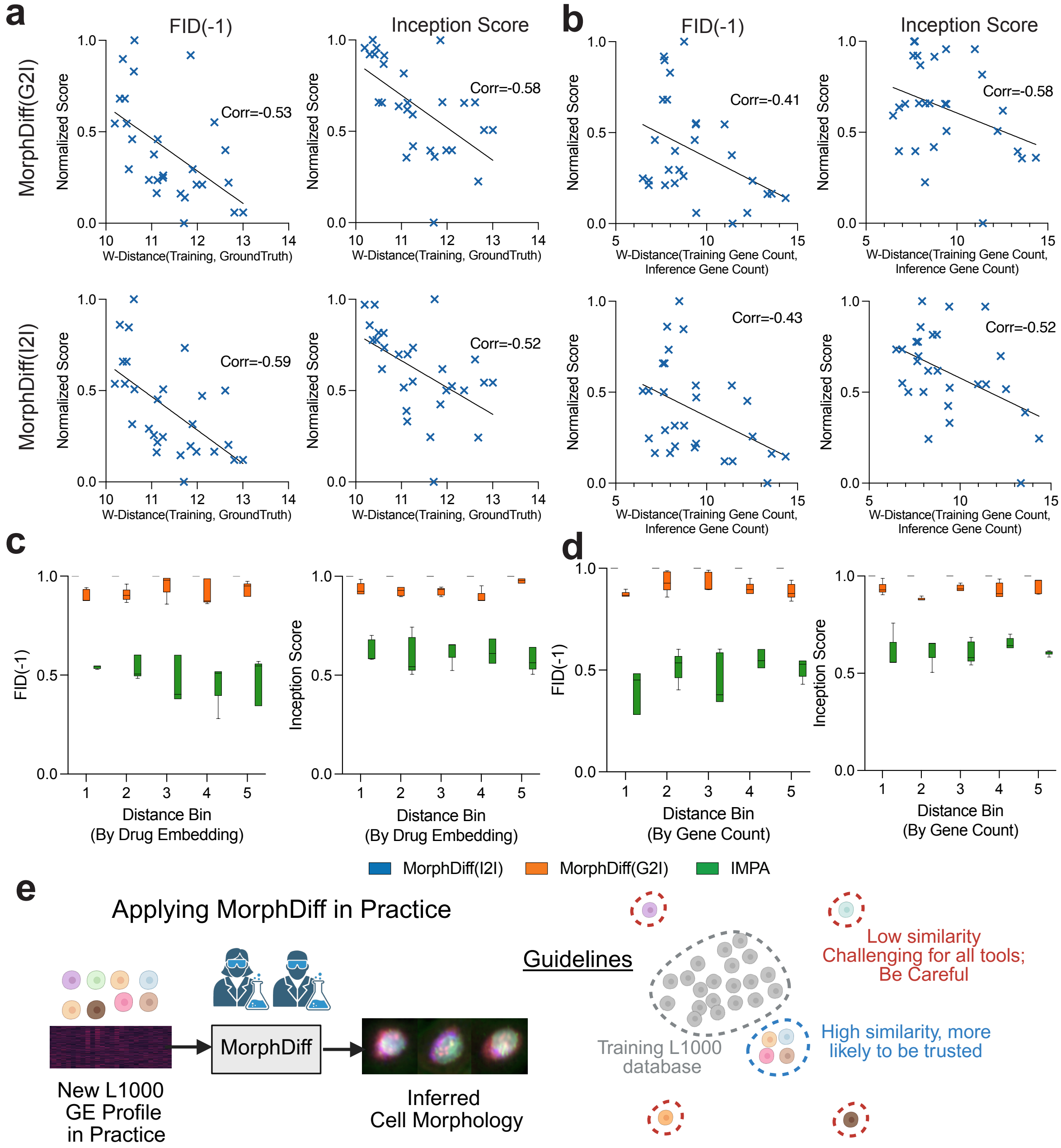

Supplementary Figure 27: Evaluating the use cases and applicability of MorphDiff. **a.** Generalization capabilities of MorphDiff(I2I) and MorphDiff(G2I) when generalizing to new MOAs on the CDRP OOD dataset. Correlation between the Wasserstein Distance of the training and ground-truth OOD data and the normalized metrics at MOA levels. The x-axis represents the Wasserstein Distance between DeepProfiler embeddings of the training and testing datasets. The y-axis displays the normalized performance metrics. The black line indicates a linear regression between the x-axis and y-axis values. **b.** Correlation between the Wasserstein Distance of training gene count and inference OOD L1000 gene count and the normalized metrics at MOA level. The x-axis represents the Wasserstein Distance between the input gene counts of the training and testing datasets. The y-axis displays the normalized performance metrics. **c and d.** Comparison of the performance of MorphDiff methods and IMPA method on the generalization capabilities with respect to the distance of drug embedding / gene count. The CDRP Target_MOA dataset were divided into five bins based on their Wasserstein Distance from the training dataset. These Wasserstein Distances were computed using gene counts and drug embeddings. All performance metrics were divided by the best performance metrics achieved on each MOA. **e.** Recommendation and practical guidelines for users of our tool. Supplementary Fig. 28-30 are extensions of this figure. Created in BioRender. Group, A. (2025) https://BioRender.com/t77r7ij. Source data are provided as a Source Data file.

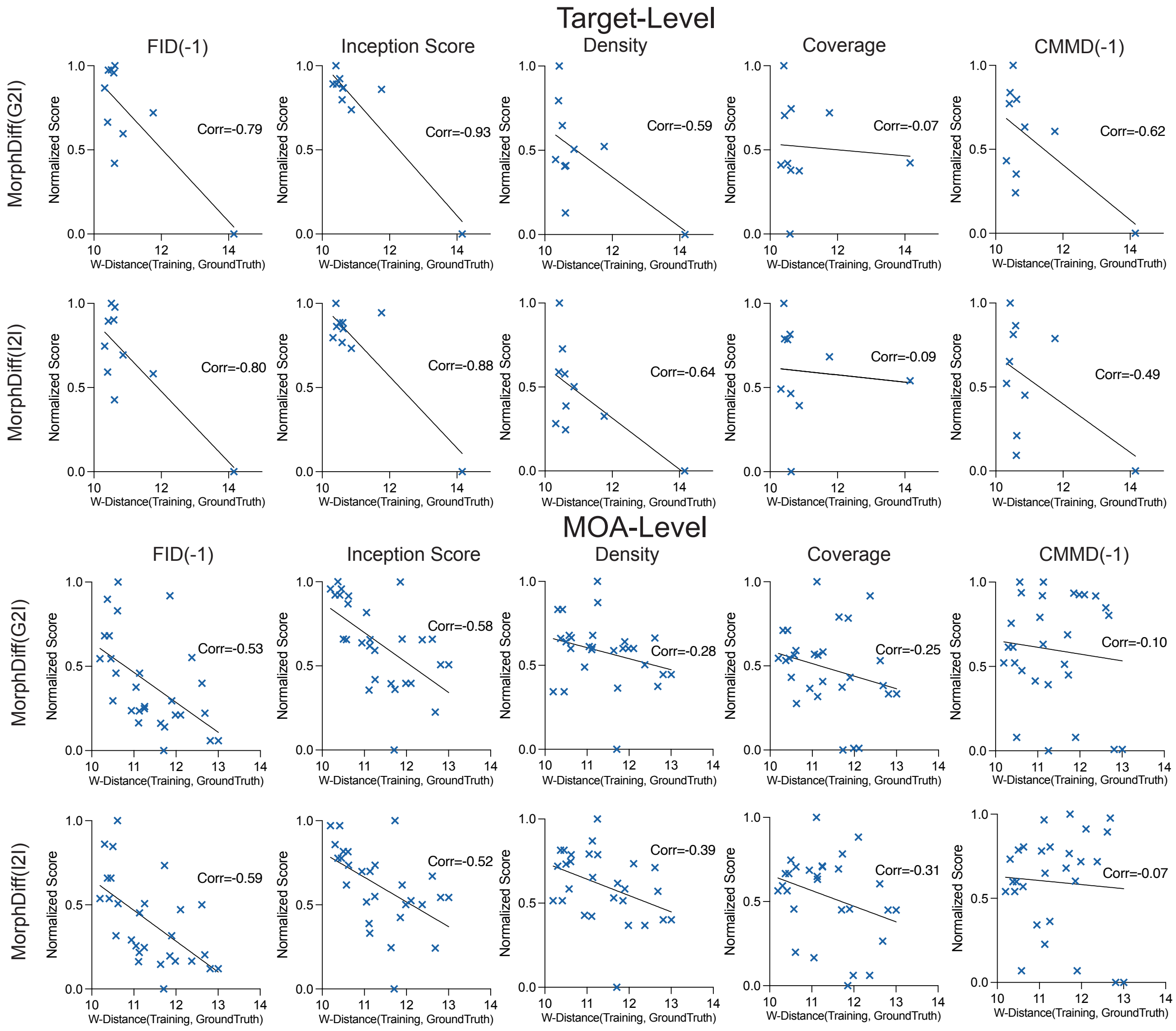

Supplementary Figure 28: Correlation between the Wasserstein Distance of the training and ground-truth OOD data and the normalized metrics at Target and MOA levels. The x-axis represents the Wasserstein Distance between DeepProfiler embeddings of the training and testing datasets. The y-axis displays the normalized performance metrics. The black line indicates a linear regression between the x-axis and y-axis values. Source data are provided as a Source Data file.

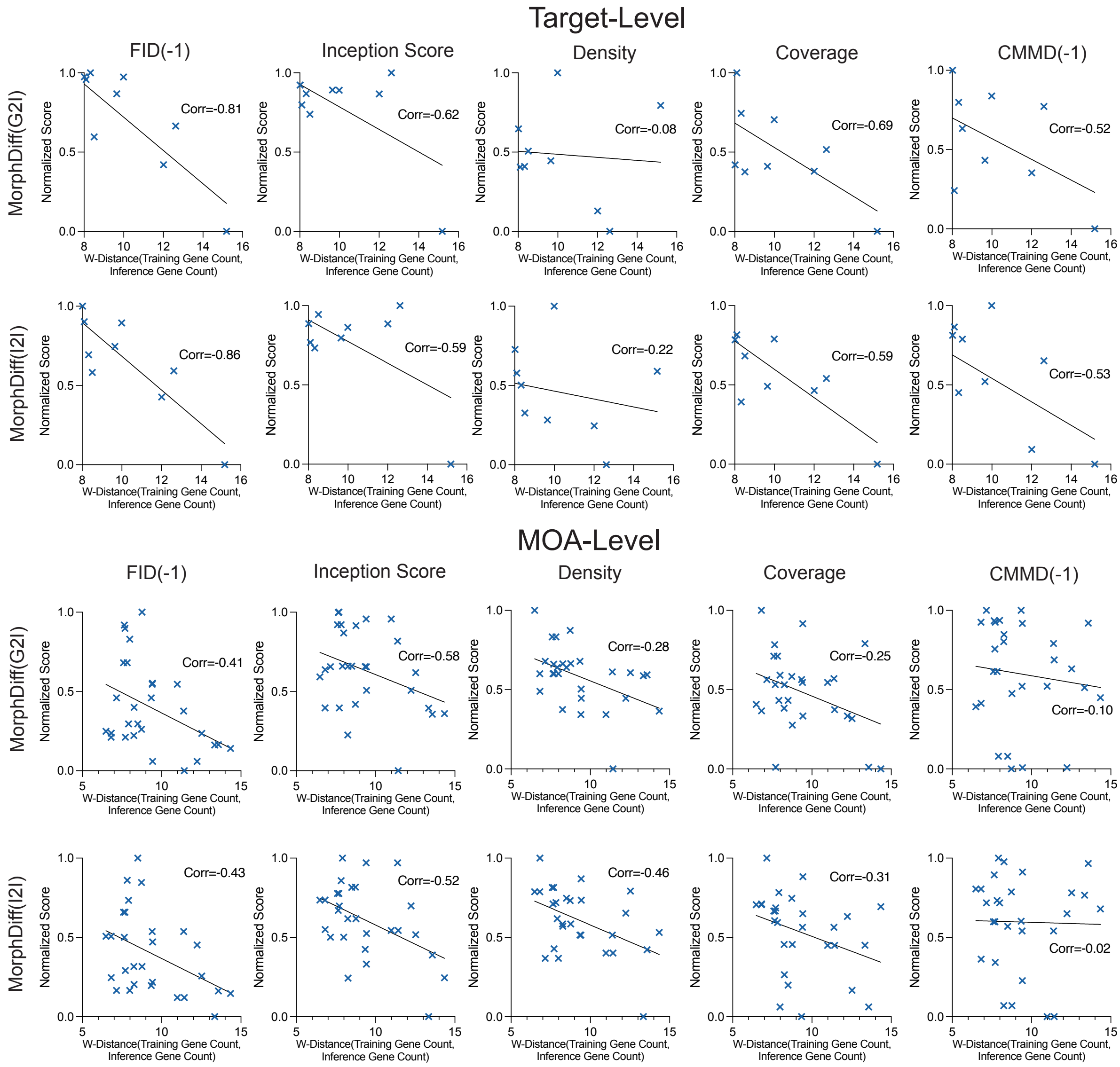

Supplementary Figure 29: Correlation between the Wasserstein Distance of training gene count and inference OOD L1000 gene count and the normalized metrics at Target and MOA levels. The x-axis represents the Wasserstein Distance between the input gene counts of the training and testing datasets. The y-axis displays the normalized performance metrics. The black line indicates a linear regression between the x-axis and y-axis values. Source data are provided as a Source Data file.

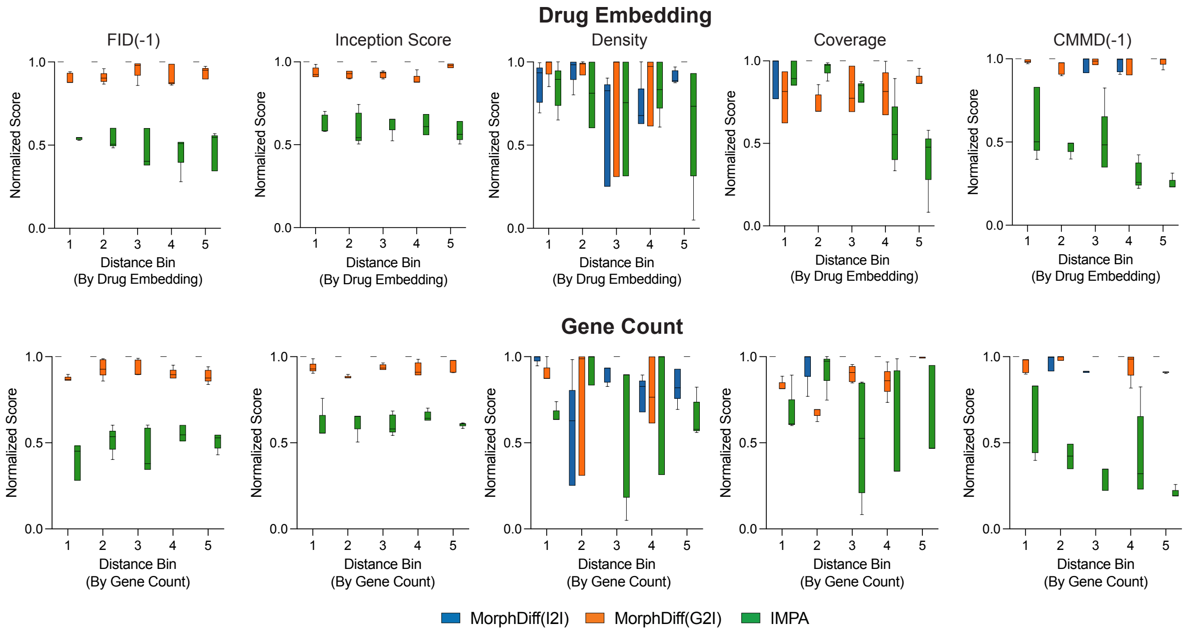

Supplementary Figure 30: Comparison of the performance of MorphDiff methods and IMPA method on the generalization capabilities with respect to the distance of drug embedding / gene count. The OOD drug MOAs were divided into five bins based on their Wasserstein Distance from the training dataset. These Wasserstein Distances were computed using gene count and drug embeddings. All performance metrics were normalized according to the best performance achieved on each MOA. Source data are provided as a Source Data file.

References

[1] Lee, H. & Welch, J. D. Morphnet predicts cell morphology from single-cell gene expression. *bioRxiv* 2022–10 (2022).

[2] Lopez, R., Regier, J., Cole, M. B., Jordan, M. I. & Yosef, N. Deep generative modeling for single-cell transcriptomics. *Nature methods* **15**, 1053–1058 (2018).

[3] Palma, A., Theis, F. J. & Lotfollahi, M. Predicting cell morphological responses to perturbations using generative modeling. *Nature Communications* **16**, 505 (2025).

[4] Choi, Y., Uh, Y., Yoo, J. & Ha, J.-W. Stargan v2: Diverse image synthesis for multiple domains. In *Proceedings of the IEEE/CVF conference on computer vision and pattern recognition*, 8188–8197 (2020).

[5] Choi, Y. *et al.* Stargan: Unified generative adversarial networks for multi-domain image-to-image translation. In *Proceedings of the IEEE conference on computer vision and pattern recognition*, 8789–8797 (2018).

[6] Lee, H.-Y. *et al.* Drit++: Diverse image-to-image translation via disentangled representations. *International Journal of Computer Vision* **128**, 2402–2417 (2020).

[7] Yu, X., Chen, Y., Liu, S., Li, T. & Li, G. Multi-mapping image-to-image translation via learning disentanglement. *Advances in Neural Information Processing Systems* **32** (2019).

[8] Esser, P., Rombach, R. & Ommer, B. Taming transformers for high-resolution image synthesis. In *Proceedings of the IEEE/CVF conference on computer vision and pattern recognition*, 12873–12883 (2021).

[9] Gao, S., Zhou, P., Cheng, M.-M. & Yan, S. Masked diffusion transformer is a strong image synthesizer. In *Proceedings of the IEEE/CVF International Conference on Computer Vision*, 23164–23173 (2023).

[10] Wang, Z., Bovik, A. C., Sheikh, H. R. & Simoncelli, E. P. Image quality assessment: from error visibility to structural similarity. *IEEE transactions on image processing* **13**, 600–612 (2004).

[11] Westfall, P. H. Kurtosis as peakedness, 1905–2014. rip. *The American Statistician* **68**, 191–195 (2014).

[12] DeCarlo, L. T. On the meaning and use of kurtosis. *Psychological methods* **2**, 292 (1997).

[13] Bunne, C. *et al.* Learning single-cell perturbation responses using neural optimal transport. *Nature methods* **20**, 1759–1768 (2023).
