# Supplementary material for: Prediction of cellular morphology changes under perturbations with a transcriptome-guided diffusion model
Source: Nat Commun. 2025 Sep 2;16:8210. doi: 10.1038/s41467-025-63478-z (PMC12405515; doi:10.1038/s41467-025-63478-z)
Supplement: Supplementary file 2 — Reporting Summary [file 41467_2025_63478_MOESM2_ESM.pdf]

nature portfolio

Corresponding author(s): Yu Li and Le Song

Last updated by author(s): Jul 23, 2025

Reporting Summary

Nature Portfolio wishes to improve the reproducibility of the work that we publish. This form provides structure for consistency and transparency in reporting. For further information on Nature Portfolio policies, see our Editorial Policies and the Editorial Policy Checklist.

Statistics

For all statistical analyses, confirm that the following items are present in the figure legend, table legend, main text, or Methods section.

n/a	Confirmed	
☐	☒	The exact sample size (n) for each experimental group/condition, given as a discrete number and unit of measurement
☐	☒	A statement on whether measurements were taken from distinct samples or whether the same sample was measured repeatedly
☐	☒	The statistical test(s) used AND whether they are one- or two-sided<br>*Only common tests should be described solely by name; describe more complex techniques in the Methods section.*
☒	☐	A description of all covariates tested
☐	☒	A description of any assumptions or corrections, such as tests of normality and adjustment for multiple comparisons
☒	☐	A full description of the statistical parameters including central tendency (e.g. means) or other basic estimates (e.g. regression coefficient) AND variation (e.g. standard deviation) or associated estimates of uncertainty (e.g. confidence intervals)
☒	☐	For null hypothesis testing, the test statistic (e.g. F, t, r) with confidence intervals, effect sizes, degrees of freedom and P value noted<br>*Give P values as exact values whenever suitable.*
☒	☐	For Bayesian analysis, information on the choice of priors and Markov chain Monte Carlo settings
☒	☐	For hierarchical and complex designs, identification of the appropriate level for tests and full reporting of outcomes
☒	☐	Estimates of effect sizes (e.g. Cohen's d, Pearson's r), indicating how they were calculated

Our web collection on statistics for biologists contains articles on many of the points above.

Software and code

Policy information about availability of computer code

Data collection	No specific software were used.
Data analysis	Tools used to analyze the data in this study include Cellprofiler v4.2.5, DeepProfiler.

For manuscripts utilizing custom algorithms or software that are central to the research but not yet described in published literature, software must be made available to editors and reviewers. We strongly encourage code deposition in a community repository (e.g. GitHub). See the Nature Portfolio guidelines for submitting code & software for further information.

Data

Policy information about availability of data

All manuscripts must include a data availability statement. This statement should provide the following information, where applicable:

- Accession codes, unique identifiers, or web links for publicly available datasets
- A description of any restrictions on data availability
- For clinical datasets or third party data, please ensure that the statement adheres to our policy

All the data used in this work is publicly available. The morphology images of CDRP dataset can be downloaded with the scripts from https://github.com/gigascience/paper-bray2017/blob/master/download_cil_images.sh. The corresponding L1000 gene expression data is collected from https://broad.io/rosetta/; The morphology images of JUMP dataset can be downloaded with the script from https://github.com/jump-cellpainting/datasets/. The corresponding L1000 gene expression data is collected from https://www.cell.com/cell/fulltext/S0092-8674(17)31309-0?dgcid=cell.com-slider_referral_cell-slider-Golub_171_6; For LINCS, the bulk morphology images are collected from https://cellpainting-gallery.s3.amazonaws.com/index.html#cpg0004-lincs/ and the corresponding gene expression data

is downloaded from https://broad.io/rosetta/. For all the genes and related pathways involved in the aforementioned genetic perturbations, as well as the drug names, SMILES (Simplified Molecular Input Line Entry System), targets (if applicable), and MOAs (if applicable) related to drug perturbations, we have provided detailed information, which is included in the Supplementary files.

Research involving human participants, their data, or biological material

Policy information about studies with human participants or human data. See also policy information about sex, gender (identity/presentation), and sexual orientation and race, ethnicity and racism.

Reporting on sex and gender	There are no human research participants in our study.
Reporting on race, ethnicity, or other socially relevant groupings	There are no human research participants in our study.
Population characteristics	There are no human research participants in our study.
Recruitment	There are no human research participants in our study.
Ethics oversight	There are no human research participants in our study.

Note that full information on the approval of the study protocol must also be provided in the manuscript.

Field-specific reporting

Please select the one below that is the best fit for your research. If you are not sure, read the appropriate sections before making your selection.

[x] Life sciences [ ] Behavioural & social sciences [ ] Ecological, evolutionary & environmental sciences

For a reference copy of the document with all sections, see nature.com/documents/nr-reporting-summary-flat.pdf

Life sciences study design

All studies must disclose on these points even when the disclosure is negative.

Sample size	We collected more than more than 300000 cell morphology images to train deep learning model. The number of images is sufficient to train deep learning model.
Data exclusions	No data is excluded from analyses.
Replication	We provide code in code ocean (10.24433/CO.4389762.v1) and GitHub for reproducibility.
Randomization	Datasets were split into random training, test splits.
Blinding	Not applicable

Reporting for specific materials, systems and methods

We require information from authors about some types of materials, experimental systems and methods used in many studies. Here, indicate whether each material, system or method listed is relevant to your study. If you are not sure if a list item applies to your research, read the appropriate section before selecting a response.

Materials & experimental systems

n/a	Involved in the study	
x		Antibodies
x		Eukaryotic cell lines
x		Palaeontology and archaeology
x		Animals and other organisms
x		Clinical data
x		Dual use research of concern
x		Plants

Methods

n/a	Involved in the study	
x		ChIP-seq
x		Flow cytometry
x		MRI-based neuroimaging

Plants

Seed stocks	Not applicable
Novel plant genotypes	Not applicable
Authentication	Not applicable
