# Supplementary material for: Prediction of cellular morphology changes under perturbations with a transcriptome-guided diffusion model
Source: Nat Commun. 2025 Sep 2;16:8210. doi: 10.1038/s41467-025-63478-z (PMC12405515; doi:10.1038/s41467-025-63478-z)
Supplement: Supplementary file 3 — Description of Additional Supplementary Files [file 41467_2025_63478_MOESM3_ESM.pdf]

Description of additional supplementary files

Supplementary Data 1

The table lists 130 genes perturbed by genetic interventions in the JUMP dataset, along with their associated pathway information from sources.

Supplementary Data 2

The table includes 959 compounds and their corresponding SMILES (Simplified Molecular Input Line Entry System) from the CDRP dataset.

Supplementary Data 3

The table lists 69 drugs, their corresponding SMILES (Simplified Molecular Input Line Entry System), and targets, annotated with 35 MOAs from the CDRP dataset.

Supplementary Data 4

The table includes 21 drugs at various dosages associated with 10 targets from the LINCS dataset.

Supplementary Data 5

The table includes 42 drugs at various dosages annotated with 10 MOAs from the LINCS dataset.

Supplementary Data 6

The table contains 8 drugs and their associated MOAs from the CDRP dataset.

Supplementary Data 7

The table contains 251 drugs and their corresponding MOAs from the CDRP dataset.
